# Differential Transcriptome Networks between IDO1-Knockout and Wild-Type Mice in Brain Microglia and Macrophages

**DOI:** 10.1371/journal.pone.0157727

**Published:** 2016-06-17

**Authors:** Dianelys Gonzalez-Pena, Scott E. Nixon, Bruce R. Southey, Marcus A. Lawson, Robert H. McCusker, Alvaro G. Hernandez, Robert Dantzer, Keith W. Kelley, Sandra L. Rodriguez-Zas

**Affiliations:** 1 Department of Animal Sciences, University of Illinois Urbana-Champaign, Urbana, Illinois, United States of America; 2 Department of Symptom Research, University of Texas M. D. Anderson Cancer Center, Houston, Texas, United States of America; 3 High-Throughput Sequencing and Genotyping Unit, Roy J. Carver Biotechnology Center, University of Illinois at Urbana-Champaign, Urbana, Illinois, United States of America; 4 Department of Statistics, University of Illinois at Urbana-Champaign, Champaign, Illinois, United States of America; 5 Carle Woese Institute for Genomic Biology, University of Illinois at Urbana-Champaign, Urbana, Illinois, United States of America; University of Florida, UNITED STATES

## Abstract

Microglia in the brain and macrophages in peripheral organs are cell types responsible for immune response to challenges. Indoleamine 2,3-dioxygenase 1 (IDO1) is an immunomodulatory enzyme of the tryptophan pathway that is expressed in the brain. The higher activity of IDO1 in response to immune challenge has been implicated in behavioral disorders. The impact of IDO1 depletion on the microglia transcriptome has not been studied. An investigation of the transcript networks in the brain microglia from IDO1-knockout (IDO1-KO) mice was undertaken, relative to peripheral macrophages and to wild-type (WT) mice under unchallenged conditions. Over 105 transcript isoforms were differentially expressed between WT and IDO1-KO within cell type. Within microglia, Saa3 and Irg1 were over-expressed in IDO1-KO relative to WT. Within macrophages, Csf3 and Sele were over-expressed in IDO1-KO relative to WT. Among the genes differentially expressed between strains, enriched biological processes included ion homeostasis and ensheathment of neurons within microglia, and cytokine and chemokine expression within macrophages. Over 11,110 transcript isoforms were differentially expressed between microglia and macrophages and of these, over 10,800 transcripts overlapped between strains. Enriched biological processes among the genes over- and under-expressed in microglia relative to macrophages included cell adhesion and apoptosis, respectively. Detected only in microglia or macrophages were 421 and 43 transcript isoforms, respectively. Alternative splicing between cell types based on differential transcript isoform abundance was detected in 210 genes including Phf11d, H2afy, and Abr. Across strains, networks depicted a predominance of genes under-expressed in microglia relative to macrophages that may be a precursor for the different response of both cell types to challenges. The detected transcriptome differences enhance the understanding of the role of IDO1 in the microglia transcriptome under unchallenged conditions.

## Introduction

During neuroinflammation microglia cells release pro-inflammatory cytokines that are associated with increased activity of indoleamine 2,3-dioxygenase (IDO), an enzyme that catalyzes tryptophan within the kynurenine pathway. Microglia are the main immune cells in the brain and, like macrophages in peripheral tissues, sense pathological events and trigger immune responses such as neuroinflammation [1]. Despite the shared overall role of brain microglia and peripheral macrophages, differences in origin, morphology and gene expression between these immune cells have been linked to distinct impact on a number of biological processes. Differential expression between these immune cells on a number of candidate genes has been reported including: genes coding for scavenger receptors [2], Toll-like receptors (Tlrs) and chemokine receptors [3], Fc receptors [4], antimicrobial peptides [5], purinergic receptors P2y and P2x [6], interferon-inducible transmembrane (Ifitms) and sialic acid—binding immunoglobulin lectins (Siglecs) [7,8]. Gene expression profiling can provide insights into the microglia population provenance and maintenance that can help understand the association between microglia and neuroinflammation.

The cascade of events elicited by the impact of neuroinflammation on IDO activity causes tryptophan depletion and quinoinic acid neural toxicity that are linked to mood disorders such as depression [9,10]. In mammals, the IDO enzyme has two homologs, IDO1 and IDO2, and IDO1 typically has significantly higher enzymatic efficiency and signaling capability than IDO2 [11]. Homozygous IDO1-knockout mice (IDO1-KO) are viable, fertile and exhibit typical immune response [12] making this strain suitable to study the role of IDO1 on molecular and physiological processes. However, no comparison of the brain microglia and peripheral macrophage transcriptome between wild type (WT) and IDO1-KO mice has been reported.

The aim of this study was to further the understanding of the role of IDO1 on the microglia transcriptome under baseline unchallenged conditions. A characterization of the microglia transcriptome of IDO1-KO mice relative to the transcriptome of peripheral macrophages and to the transcriptome of WT mice in the absence of experimental immune challenge was undertaken. This first step to gain insights into the transcriptome profile associated with IDO1 was accomplished by: 1) profiling the transcript isoforms and detecting differential alternative splicing and gene expression between microglia and macrophages within IDO1-KO and WT mouse strains; 2) profiling the transcript isoforms and detection of differential alternative splicing and gene expression between IDO1-KO and WT mouse strains within microglia and macrophages; and 3) using networks to uncover potential synergistic or antagonistic relationships between cell types and mouse strains.

## Materials and Methods

### Ethics statement

All procedures were conducted following the guidelines recommended in the Guide for the Care and Use of Laboratory Animals of the National Institute of Health with the approval of the University of Illinois Institutional Animal Care and Use Committee.

### Animals, Experimental Design, Cell Isolation, and RNA Extraction

Adult (~22 weeks old) male mice from two strains: WT (C57BL/6J) and IDO1-KO (C57Bl/6J background) were studied. Mice (n = 12/strain) were housed in individual cages under a normal 12:12 h light/dark cycle in a temperature- (23°C) and humidity- (45%) controlled room. Mice were offered water and food *ad libitum* (Teklad 8640 chow, Harlan Laboratories, Indianapolis, IN, USA). Mice were not exposed to experimental immune challenges. Trained personnel euthanized the mice by CO_2_ asphyxiation and all efforts were made to minimize suffering.

Peritoneal macrophages (subsequently denoted macrophages) were collected from peritoneal tissue using the standards protocols [13,14]. Abdomens were disinfected with alcohol 70% post-euthanasia and an incision to retract skin was made. The peritoneal cavity was flushed with 10 ml of Hank's Balanced Salt Solution (HBSS, cold harvest medium) and the recovery peritoneal fluid was centrifuged at 400 g. The cell pellet was resuspended in warm Dulbecco's Modified Eagle's Medium-10 (DMEM supplemented with 10 mM glutamine, 100 μg/ml penicillin and streptomycin) medium and plated. After a 2-h incubation at 37°C with 5% CO_2_, the medium was changed to remove non-adherent cells. The surviving adherent cells constituted the non-thioglycollate elicited peritoneal macrophages and cells from individual mice were stored in Trizol at -80°C until RNA extraction [15].

Immediately following the collection of macrophages, mice were perfused, and the brains were collected for microglia isolation [16]. Briefly, microglia samples were extracted from dissected, minced, and trypsinized brains [17]. Tissue debris was eliminated using nylon cell strainers (40 μm). Cells were centrifuged 1,000 g for 10 minutes for myelin removal and resuspended in 30% Percoll (GE Healthcare, Princeton, NJ). Cells were labeled with anti-CD11b antibodies (Miltenyi Biotec, Germany) in PEB buffer (PBS supplemented with 0.5% bovine serum albumin and 2 mM Ethylenediaminetetraacetic acid) for 15 minutes. Labeled cells were incubated for 15 minutes with anti-CD11b magnetized microbeads (Miltenyi Biotec, Germany). CD11b^+^ cells were separated in a magnetic field using MS columns for positive selection (Miltenyi Biotec, Germany). Microglia from individual mice was pelleted and resuspended in Trizol at -80°C for RNA extraction [15].

Resting microglia in rodents is characterized by low expression of CD45 and thus a dual marker phenotype CD11b^+^ and CD45^low^ was used to confirm microglia isolation meanwhile a phenotype CD11b^+^ and CD45^high^ was used to confirm peripheral macrophage isolation [18,19]. Flow cytometry validation of cell isolation encompassed cell staining with primary fluorescent antibodies CD11b and CD45 [18,19]. Incubation with anti-CD16/CD32 antibody before incubation with eBioscience anti-CD11b and anti-CD45 antibodies enabled blocking Fc receptors (http://www.ebiosciencecom). Surface receptor expression was detected using a Biosciences LSR II Flow Cytometry Analyzer with BD FACSDiva software (http://www.bdbiosciences.com). Antibody gating was determined using isotype-stained controls. This flow cytometry analysis revealed that microglia and macrophage isolation yielded more than 85% and 80% population purity, respectively.

RNA extraction using the Total RNA Kit (Omega Biotek, Norcross, GA) applied steps from the Trispin method including an extraction column from the E.Z.N.A., and a DNase step to remove DNA contamination [16]. RNA Integrity Numbers (RIN) were determined using the Agilent 2100 Bioanalyzer with RNA Pico chip (Agilent Technologies, Palo Alto, CA). This indicator was > 9 in 90% of the samples and > 7 in all the samples.

### Differential Expression Analysis

Individual mouse RNA-Seq libraries for microglia and macrophages were sequenced using Illumina HiSeq2000 (Illumina, San Diego, CA). The quality of the resulting 100 nt paired-end read FASTQ files was assessed using FastQC (Babraham Institute, 2013). The paired-end reads were mapped to the C57Bl/6J mouse genome (version GRCm38) using TopHat v2.0.6 [20] and default settings.

The mapped reads were assembled into transcript isoforms and differentially expressed transcript isoforms among strains and cell types were identified using Cuffdiff v2.1.1 within the Galaxy environment [20,21]. Cuffdiff settings were: minimum alignment of five reads per transcript isoform for differential abundance testing; quartile normalization to minimize the impact of extreme read counts by removing the top 25% from each library; fragment bias correction to accommodate over- or under-representation of fragments due to sequence-specific or positional bias, and multi-read correction to weigh the contribution of a read that maps to multiple locations. Cuffdiff assumes a negative binomial distribution when comparing expression levels between strain and cell type groups.

Four complementary comparisons of transcript isoforms and genes were performed: 1) microglia IDO1-KO vs. microglia WT; 2) macrophage IDO1-KO vs. macrophage WT; 3) microglia IDO1-KO vs macrophage IDO1-KO; and 4) microglia WT vs. macrophage WT. A false discovery rate (FDR) was used to adjust for multiple testing [22,23]. Transcript isoforms and genes were considered differentially expressed at FDR-adjusted P-value < 0.05.

### Profile validation

Validation of gene detection and differential expression across cell types and strains followed two approaches. Detection of genes was confirmed at the protein level using results from tandem mass spectrometry analysis of the shared samples [24,25]. Briefly, proteins in the samples were detected using a bottom-up proteomics approach including trypsin digestion followed by peptide separation using High Performance Liquid Chromatography (HPLC) and tandem mass spectrometry using Thermo LTQ-FT LC/MS/MS (Thermo Fisher Scientific; http://www.thermofisher.com). Proteins identification was accomplished using the database search program PEAKS Studio v7 (Bioinformatics Solutions Inc.; http://www.bioinfor.com). Confirmation of differential expression between cell type and strain groups relied on a three-fold cross-validation approach. The complete data set was randomly divided into three independent subsets (n = 4 mice/strain/subset). Each data subset was analyzed applying the same model used in the analysis of the complete data set and differential gene expression was compared across analyses.

### Alternative Splicing and Transcript Detection

Qualitative and quantitative characterization of alternative splicing between strains and cell types was undertaken [26]. The criterion for qualitative characterization of alternative splicing differentiating cell types or strains consisted on detecting genes or transcript isoforms in one strain or cell type group that were undetected (zero mapped reads) in the other strain or cell type group. The criterion for quantitative characterization of alternative splicing was at least one form differentially expressed (FDR-adjusted P-value < 0.05) between cell types or strains in either direction (over or under-expressed in microglia or in WT) and other form(s) differentially expressed in the opposite direction or not differentially expressed. The length and relative location within the gene of the transcript isoforms differentially expressed between cell types was visualized using the ENSEMBL genome browser [27].

### Functional and Gene Network Analyses

Complementing the study of individual genes, functional analyses of transcript isoforms exclusively detected in one strain or cell type group or differentially expressed between strains within cell type and between cell types within strain were performed using the Database for Annotation, Visualization and Integrated Discovery (DAVID) [28,29]. These analyses allowed the identification of Gene Ontology (GO, http://www.geneontology.org/) biological processes, molecular functions, and Kyoto Encyclopedia of Genes and Genomes (KEGG) pathways (http://www.genome.jp/org/) enriched among the gene lists. Gene Ontology results were reported using the DAVID classification of terms Functional Annotation Tool (FAT) to optimize the information offered by the categories reported. Evidence of enrichment was represented using Expression Analysis Systematic Explorer (EASE) scores computed based on a one-tailed jackknifed Fisher exact test [30]. Interpretation was further facilitated using the DAVID functional annotation clustering of categories. The statistical significance of each functional cluster was assessed using an Enrichment Score computed as the geometric mean (on the -log_10_ scale) of the EASE scores of the categories in a cluster [21,31]. Functional annotation clusters were considered significant at Enrichment Score > 2 (comparable to a cluster P-value < 0.001) using the *Mus musculus* genome as background for testing.

Confirmatory and complementary Gene Set Enrichment Analysis (GSEA) was implemented using the GSEA-P software package [32]. The complementarity of GSEA relies on the use of the complete list of gene profiles that exploits gene dependencies and the annotation of functional categories in the Molecular Signature Database.

Interpretation of findings and discovery of differences in gene relationships between cell types within strain and between strains within cell type was facilitated using network reconstruction. Protein-protein interactions and transcription factors associated with differential expression between cell types were studied using the STRING 9.1 software [33]. The thickness of the edges connecting genes or nodes indicates the strength of the known relationships between proteins. A second set of gene networks were created and visualized within the Cytoscape environment [34,35] using the BisoGenet plug-in [36,37]. Only gene-gene interactions that had at most one neighbor connecting the genes were considered and the edges denote known relationships between genes summarized in the SysBiomics repository. Estimates of expression differences between cell types or strains and associated P-values from our study were integrated. Differentially expressed genes (also termed target genes) detected in the present study and intermediate connecting genes were represented by nodes and their known relationships by edges. The node size represent the differential expression P-value with larger nodes indicating more significant values and the colors denote over- or under-expression between cell types or strains.

## Results and Discussion

### Alignment of Sequence Reads

The number of sequence reads within strain and cell type library was on average 60 million pairs. On average, 90% of the reads were mapped to the mouse genome, 85% of the reads were mapped in pairs, and 76% of the reads were mapped to a single genome location (**Table A** in S1 File). The consistency on read mapping across strains and cell types was also observed in the distribution of the expression levels among the 28,403 annotated genes (**Fig A** in S1 File). Considering transcript isoforms represented by at least five reads and using the indicator of abundance the fragments per kilobase of transcript per million reads in the library (FPKM), more than 64% of the annotated genes exhibited FPKM > 0 and less than 0.1% of the annotated genes showed expression levels beyond 1000 FPKM within group. These values are consistent with reports from similar RNA-Seq studies in mice [13,21] and confirms that all strain and cell type samples were sequenced with adequate and comparable depth. These results also confirm that the cell isolation protocols did not have substantial effect on the range of transcriptome profiles.

### Global Transcriptome Profiles across Cell Types and Strains

**Fig 1:**
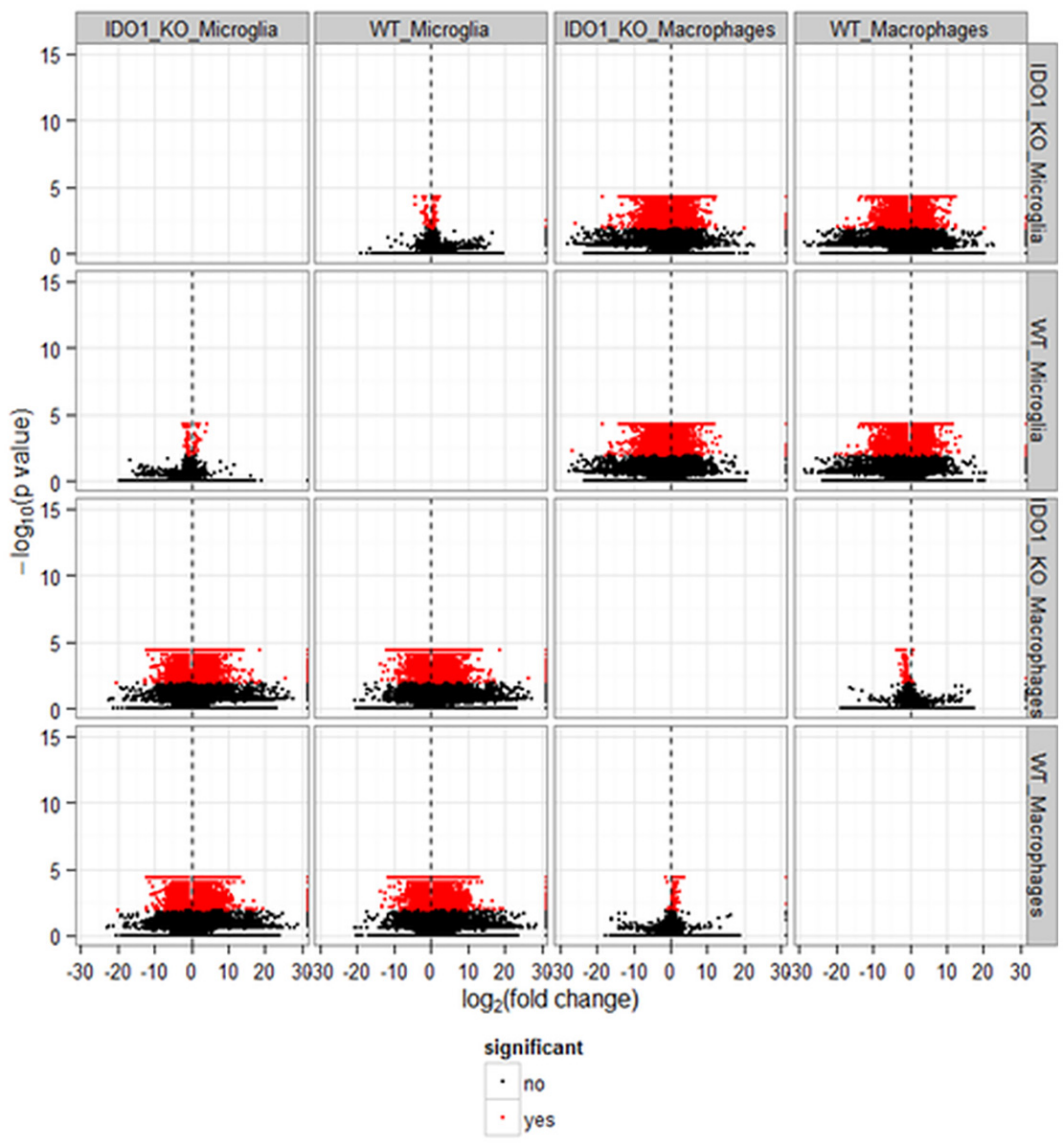
Volcano plots of transcript isoform differential expression between microglia and macrophage cells within strain and between IDO1-knock out (IDO1_KO) and wild type (WT) strains within cell type expressed in -10log10(P-value) in the y-axis versus log2(fold change) in the x-axis, where fold change is computed between the pair of cell type-strain combination labels. Red dots denote transcript isoforms differentially expressed at FDR-adjusted P-value < 0.05 (labeled significant yes) and black dots denote transcript isoforms not differentially expressed (labeled significant no).

The number of differentially expressed genes and transcript isoforms was dominated by differences between microglia and macrophages relative to differences between IDO1-KO and WT strains (Table 1). On average, 9,908 genes were differentially expressed between cell types within strain and 120 genes were differentially expressed between strains within cell types (Fig 1). In consideration of the 100 fold higher differential expression between cell types relative to strains, results of the cell type comparison are presented first.

**Table 1:** Number of genes and transcript isoforms analyzed and differentially expressed between microglia and macrophages within and between IDO1-knockout (KO) and wild type (WT) mice.

Comparison	Gene		Transcripts Isoform	
	Analyzed	DE or Unique	Analyzed	DE or Unique
Microglia IDO1-KO vs. Microglia WT	14,981	103	27,880	108
Macrophages IDO1-KO vs. Macrophage WT	13,074	137	24,813	144
Overlap between previous two comparisons		8		10
Microglia IDO1-KO vs. Macrophages IDO1-KO	15,237	9,956	28,674	11,423
Microglia WT vs. Macrophages WT	15,287	9,860	29,110	11,113
Overlap between previous two comparisons		9,293		10,865
Unique to Macrophage WT		23		42
Unique to Microglia WT		236		421
Unique to Macrophage IDO1-KO		16		45
Unique to Microglia IDO1-KO		221		358

DE^1^: differentially expressed genes or transcript isoforms (FDR-adjusted P-value < 0.05) or uniquely detected in either cell type.

### Validation

The three-fold cross-validation of the differentially expressed genes between microglia and macrophages within strain confirmed that 87% of the genes were significantly differentially expressed at the same stringent differential expression threshold used for the complete data set. The three-fold cross-validation of genes differentially expressed between strains confirmed 71% of the genes in macrophages and 56% of the genes in microglia. Furthermore, more than 70% of the proteins detected by tandem mass spectrometry analysis were tested in the transcriptomic analysis.

The stronger validation of differential gene expression between cell types relative to strains is consistent with the higher number of extreme differentially expressed genes between cell types relative to strains. This finding is also consistent with a prior comparison between immune cell types [7] and with reports of similar physiology, mechanical allodynia and thermal hyperalgesia between IDO1-KO and WT mice under unchallenged conditions [12].

### Transcriptomic Comparison between Microglia and Macrophages within Strain

Differences in gene expression between microglia and macrophages identified in both strains are listed in Table 1. These differences are consistent with prior studies [3,7,38] and have been attributed to the origin [38] and local environment [7] differences. The consistency in overall gene expression levels between cell types in this study and with prior studies indicates that the cell isolation protocol used in this study did not have a major impact on the transcriptome.

The major overlap in differentially expressed genes (> 92%) and transcript isoforms between microglia and macrophages and relatively fewer genes only detected in a single cell type across strains suggests conservation of cell function between WT and IDO1-KO strains (Table 1). Furthermore, the overarching common role of macrophages and microglia, sometimes described as resident macrophage-like cells is confirmed by the substantial number of expressed genes and transcript isoforms that do not exhibit differential expression between cell types. Non-differential expression was assessed among 35% of the expressed genes and 60% of the transcript isoforms in IDO1-KO and among 35% of the expressed genes and 62% of the transcript isoforms in WT mice (Table 1). Similarities in gene expression between microglia and macrophages were also reported among the top 10% expressed transcript isoforms in 5 and 24 month-old mice [7].

The number of expressed genes detected solely in the microglia and macrophages of IDO1-KO mice was 221 and 16, respectively (Table 1). The number of expressed genes detected solely in the microglia and macrophages of WT mice was 236 and 23, respectively. This result is consistent with previous reports of genes expressed uniquely in the microglia [7]. Genes solely detected in macrophages or microglia in IDO1-KO constituted 75% and 93% of the corresponding expressed genes detected in WT, respectively.

Our study confirmed a number of microglia-specific expressed genes that are considered microglia signatures (**Table B** in S1 File) [39]. These genes included F11 receptor (F11r), olfactomedin-like 3 (Olfml3), G protein-coupled receptor 56 (Gpr56), Serine (or cysteine) peptidase inhibitor clade E member 2 (Serpine2) [40], calcium/calmodulin-dependent serine protein kinase (Cask) [41], and sialic acid binding Ig-like lectin H (Siglech). Furthermore, the profiles of the transcripts corresponding to the antibodies used to discriminate microglia from macrophages were consistent with the expected antibody profiles. Integrin alpha M (Itgam or Cd11b) was expressed both in cell types across strains, and protein tyrosine phosphatase, receptor type, C (Ptprc or CD45) was differentially over-expressed in macrophages relative to microglia in both mouse strains (FDR-adjusted P-value < 0.0002). The substantial overlap between strains in differential expression profiles between cell types and also in cell type-specific genes suggests that these findings are associated with functional differences between the cell types.

The 20 most extreme differentially expressed genes between microglia and macrophages in WT (|log_2_(fold change)| > 6, FDR-adjusted P-value < 5.0 x 10^−5^) are listed in Table 2 together with supporting literature. An extended list of differentially expressed genes is provided within **Table B** in S1 File.

**Table 2:** Most extreme differentially expressed genes (|log_2_(fold change)| > 6, FDR-adjusted P-value < 5.0 x 10^−5^) between microglia and macrophages in wild type mice.

Gene Symbol	Gene Name	Log_2_(Macrophage/Microglia)	Literature Review Reference
Slc2a5	solute carrier family 2 (facilitated glucose transporter), member 5	-12.95	[7,42]
Gpr56	G protein-coupled receptor 56	-12.50	[7,42]
Olfml3	olfactomedin-like 3	-12.45	[7,42]
Siglech	sialic acid binding Ig-like lectin H	-11.64	[7,42]
Crybb1	crystallin, beta B1	-10.47	[7,42]
S100a9	S100 calcium binding protein A9 (calgranulin B)	-10.02	[7]
Cask	calcium/calmodulin-dependent serine protein kinase	-9.77	[41]
Serpine2	serine (or cysteine) peptidase inhibitor, clade E, member 2	-8.60	[40,42]
Ang	angiogenin, ribonuclease, RNase A family, 5	-8.37	[42]
Hpgd	hydroxyprostaglandin dehydrogenase 15 (NAD)	-7.65	[7]
Rab3il1	RAB3A interacting protein (rabin3)-like 1	-7.49	[42]
Fosb	FBJ osteosarcoma oncogene B	-7.31	[43]
Lag3	lymphocyte-activation gene 3	-7.25	[42]
S100a8	S100 calcium binding protein A8 (calgranulin A)	-7.20	[7]
F11r	F11 receptor	-6.95	[42]
Fscn1	fascin homolog 1, actin bundling protein	-6.16	[42]
Wfdc17	WAP four-disulfide core domain 17	6.38	[44]
Cxcl1	chemokine (C-X-C motif) ligand 1	7.33	[3]
Alox15	arachidonate 15-lipoxygenase	8.05	[7]
Ccl24	chemokine (C-C motif) ligand 24	8.09	[45]
Clec4d	C-type lectin domain family 4, member d	8.45	[46]
Retnla	resistin like alpha	9.29	[7]
Olr1	oxidized low density lipoprotein (lectin-like) receptor 1	10.00	[47]
Lyz1	lysozyme 1	10.89	[48]
Cd5l	CD5 antigen-like	11.13	[7]
Cxcl13	chemokine (C-X-C motif) ligand 13	11.75	[7]

A number of genes differentially expressed between microglia and macrophages in this study have been associated with neuropsychiatric disorders, thus confirming the role of microglia on behavior and cognition [49]. Significantly over-expressed genes in microglia relative to macrophages included: Cask, Rpgrip1-like (Rpgrip1l) transmembrane protein 67 (Tmem67) and myelin protein zero (Mpz) that have been previously identified in neuropsychiatric disorders [50–53]. The expression profiles of these genes were also consistent with neurotoxic or neuroprotective impact of microglia phenotypes [7]. Classical microglia activation or M1 microglia phenotype contributes to neuronal damage by releasing pro-inflammatory cytokines, reactive oxygen species, and proteinases [54]. Deactivated, non-classically activation, or M2 microglia phenotype maintains tissues integrity and promotes axonal elongation [54]. Our results provide evidence supporting the continuum of the M1/M2 paradigm in the microglia under baseline unchallenged conditions.

The triggering receptor expressed on myeloid cells 2 (Trem2), an innate immune receptor that regulates microglial cytokine production and phagocytosis of apoptotic neurons without eliciting inflammation [55], is associated with neurodegenerative diseases such as Alzheimer’s disease (**Table B** in S1 File) [56]. Trem2 stimulation induces phosphorylation of DNAX-activating protein of molecular mass 12 kDa (DAP12), an important regulator of the microglial transcriptome [7]. Siglech, a microglia signature gene [57] that interacts with DAP12, was also over-expressed in microglia relative to macrophages (Table 2) [58].

Additional genes that were over-expressed in the microglia relative to macrophages included transcription factors that participate in the Toll-like receptor signaling pathway (e.g. FBJ Murine Osteosarcoma Viral Oncogene Homolog B or FosB), carbohydrate-binding receptors and domain containing molecules (e.g. Siglech, F11r), and cytokine-binding receptors (e.g. transforming growth factor, beta receptor I). The functions of these genes are consistent with the scavenging and innate immune function of the microglia cells [8,59]. Microglia elicits M1 pro-inflammatory cytotoxic or M2 anti-inflammatory repair-promoting response as a function of the carbohydrate-binding receptors activated. Microglia phagocytosis under potential neurodegenerative or neuroinflammatory processes is also regulated by carbohydrate-binding receptors. Our results provide novel evidence supporting the prior hypothesis that microglia can achieve equilibrium in carbohydrate-binding receptor signaling through immunoreceptor tyrosine-based activation motif or an immunoreceptor tyrosine-based inhibitory motif [8].

Several genes under-expressed in microglia relative to macrophages participate in immune response including chemokines (C-X-C motif) ligand 13 (CxCl13); C-X-C motif ligand 1 (Cxcl1); members of the chemokine signaling pathway (C-C motif) ligand 24 (Ccl24); immune response C-type lectin domain family 4 member d (Clec4d); a negative regulator of the adaptive immune response arachidonate 15-lipoxygenase (Alox15); WAP four-disulfide core domain 17 (Wdfc17); and antimicrobial properties like lysozyme 1 (Lyz1). The under-expression of chemokine genes in the microglia offer protection against neurotoxic effects. The detection of only one marker of macrophage activation (Wfdc17) [44] although not among the top differentially expressed genes confirms the unchallenged experimental conditions. The detection of Lyz1 was expected because lysozymes are one of the most abundant antimicrobial proteins in the airspaces of the lung [60]. Likewise, Alox15 products exhibit anti-inflammatory activities [61], preventing the activation of the innate immune response [62].

### Functional Analysis of the Transcriptomic Comparison between Microglia and Macrophages within Strain

A number of GO and KEGG categories were enriched among the genes differentially expressed between microglia and macrophages using DAVID. Table 3 summarizes the clusters of functional categories that have Enrichment Score > 3 and **Table C** in S1 File presents the complete list of categories that have Enrichment Score > 2 and corresponding gene counts.

**Table 3:** Enriched (DAVID Enrichment score ES > 2) functional clusters of Gene Ontology (GO) biological processes (BP) and molecular functions (MF) encompassing transcript isoforms differentially expressed between microglia and macrophages in wild type mice.

Cluster^1^	Representative GO Terms^2^	ES
BP	cell adhesion; biological adhesion; cell-cell adhesion	11.85
MF	carbohydrate binding; pattern binding; polysaccharide binding	8.07
BP	response to wounding; inflammatory response; defense response	6.67
BP	cell motion; cell projection organization; neuron projection development	5.93
BP	cell motion; cell migration; localization of cell	5.08
MF, BP	GTPase regulator activity; nucleoside-triphosphatase regulator activity; regulation of small GTPase mediated signal transduction	4.60
BP	vasculature development; blood vessel development; blood vessel morphogenesis	3.73
BP	taxis; chemotaxis; locomotory behavior	3.72
BP	regulation of leukocyte proliferation; regulation of leukocyte activation; positive regulation of immune system process	3.68
MF	cytokine activity; chemokine activity; chemokine receptor binding	3.3
BP	positive regulation of immune system process; positive regulation of response to stimulus; immune response-regulating signal transduction	3.26
MF	guanyl ribonucleotide binding; guanyl nucleotide binding; GTP binding	3.12

^1^ Each row corresponds to a cluster of Functional Annotation Tool (FAT) GO categories.

^2^ The three GO terms exhibiting most significant enrichment P-value within each cluster are listed, separated by “;”. Additional information in each cluster is provided in S3 Table.

The enriched functional categories offer insights into differences between microglia and macrophages (Table 3). The enriched categories are consistent with previous studies of murine microglia and included regulation of immune system process; cytokine and chemokine activity; and scavenging [7].

Functional analysis using GSEA confirmed the enrichment results from DAVID and enabled the discrimination of enrichment among over- and under-expressed transcript isoforms between microglia and macrophages. All except one category detected as enriched in the GSEA analysis were under-expressed in microglia relative to macrophages in WT at FDR-adjusted P-value < 0.05 that have a minimum of 15 genes within category (Table 4). **Table D** in S1 File includes categories enriched at FDR-adjusted P-value < 0.05 that have a minimum of 10 genes within category. The under-expression of genes in categories associated with immune response, ribosome and signaling in the microglia relative to macrophages is related to the detrimental neurological effect of microglia M1 activation in a potential immune challenge. Lower levels of key transcript isoforms may contain the effects of microglia activation against potential innocuous challenges and avoid negative side effects, particularly to neural processes.

**Table 4:** Enriched Gene Set Enrichment Analysis categories (FDR-adjusted P-value < 0.01and > 10 transcript isoforms within category) encompassing transcript isoforms under-expressed in microglia relative to macrophages.

Category	NG^1^	FDR^2^
KEGG_ribosome	64	<0.00E-05
Structural_Constituent_of_Ribosome	55	<0.00E-05
KEGG_Hematopoietic_Cell_Lineage	46	<0.00E-05
Immune_Response	123	<0.00E-05
KEGG_Primary_Immunodeficiency	19	<0.00E-05
Immune_System_Process	175	3.27E-03
Locomotory_Behavior	47	5.08E-03
Chemokine_Receptor_Binding	20	5.28E-03
Chemokine_Activity	20	7.10E-03
Cytokine_Activity	43	7.61E-03
Translation	98	7.40E-03

^1^ NG: number of genes in each enriched category.

^2^ FDR: adjusted P-value.

### Cell Type-Specific Alternative Splicing Events and Transcript Isoforms

Beyond the identification of differentially expressed and cell type-specific genes, transcript isoforms resulting from alternative splicing were investigated to understand the functional differences between microglia and macrophages. **Table E** in S1 File lists all the genes that match the broader quantitative alternative splicing definition of at least one transcript isoform differentially expressed among multiple isoforms. Our analysis identified 210 genes (including 826 transcript isoforms) exhibiting alternative splicing identified by at least one transcript isoform differentially expressed between microglia and macrophages among multiple isoforms detected for the gene. In addition, 237 differentially expressed (FDR-adjusted P-value < 0.05) transcript isoforms corresponded to loci that were not differentially expressed between cell types at the gene level. A number of genes encompassing differentially expressed transcript isoforms were associated with microglia, including α subunit of LFA-1(αLβ2) integrin (Itgal or Cd11a) that is highly expressed in microglia and controls the migration of these cells to injury locations and protects against ischemic brain damage [63]. Our transcriptome-wide findings confirmed reports of individual alternative splicing events in the same cell types including an alternative splicing product of the A4-amyloid precursor protein (APP) associated with Alzheimer’s disease [64]. Also, Autism Spectrum Disorder has been associated to a number of alternative spliced genes affiliated to macrophage-related pathways in the brain [65]. Table 5 summarizes genes with at least four transcript isoforms and at least two differentially expressed (FDR-adjusted P-value < 0.05) between microglia and macrophages.

**Table 5:** Genes exhibiting an alternative splicing event between microglia and macrophages in wild type mice including at least four transcript isoforms and at least two over- or under-expressed (FDR-adjusted P-value < 0.05) transcript isoforms between cell types.

Gene Symbol	Gene Name	Differential Expression		
		Over^1^	Under^2^	Non^3^
H2afy	H2A histone family, member Y	2	2	3
Foxj1	Forkhead box J1	0	2	2
Phf11	PHD finger protein 11	2	1	1
Abr	Active BCR-related gene	1	1	3
Arhgap17	Rho GTPase activating protein 17	1	1	7
Clk1	CDC-like kinase 1	1	1	2
Elf2	E74-like factor 2	1	1	5
Fam132b	Family with sequence similarity 132, member B	1	1	4
Gas2l1	Growth arrest-specific 2 like 1	1	1	4
Hmox2	Heme oxygenase (decycling) 2	1	1	3
Ndst1	N-deacetylase/N-sulfotransferase (heparan glucosaminyl) 1	1	1	2
Rbm5	RNA binding motif protein 5	1	1	4
Slc25a25	Solute carrier family 25 (mitochondrial carrier, phosphate carrier), member 25	1	1	2
Slc4a7	Solute carrier family 4, sodium bicarbonate cotransporter, member 7	1	1	6
Zfp869	Zinc finger protein 869	1	1	7
Hivep3	Human immunodeficiency virus type I enhancer binding protein 3	2	0	3
Rad52	RAD52 homolog	2	0	4

^1^ Over: transcript isoforms over-expressed in macrophage

^2^ Under: transcript isoforms under-expressed in macrophage

^3^ Non: not differentially expressed transcript isoforms (FDR-adjusted P-value < 0.05)

**Fig 2:**
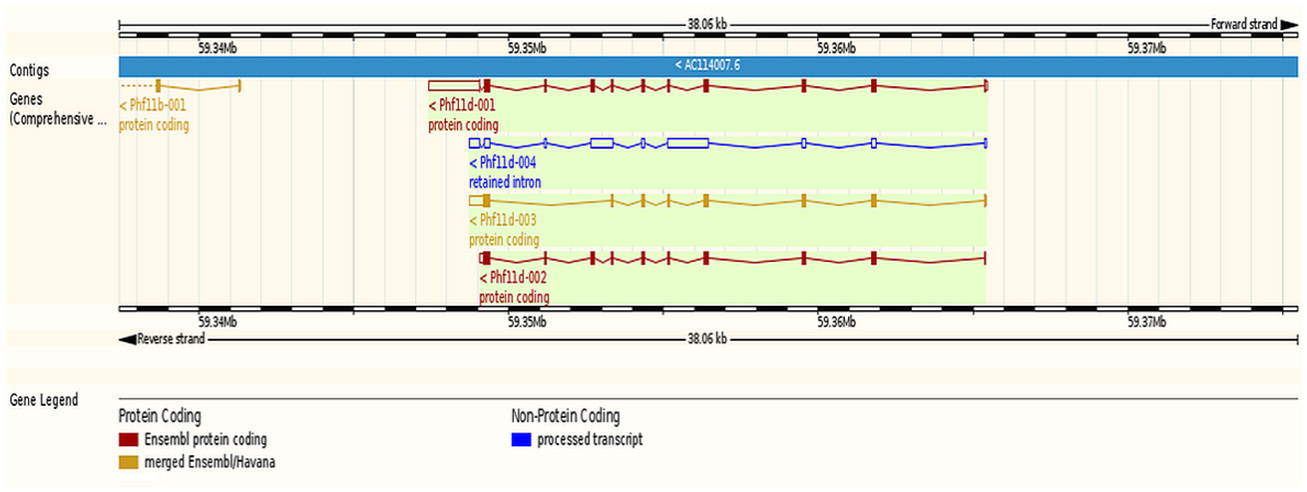
Relative location of the PHD finger protein 11 d (Phf11d) transcript isoforms associated with an alternative splicing event between microglia and macrophages (Ensembl, http://www.ensembl.org). Red denotes Ensembl protein coding, orange denotes merged Ensembl/Havana protein coding, and blue denotes processed transcript non-protein coding.

Three genes exhibiting at least one differentially expressed transcript isoform between microglia and macrophages in this study are highlighted because of independent studies supporting their promise to understand microglia and macrophage differences. These genes are PHD finger protein 11 d (Phf11d), histone family, member Y (H2afy), and active BCR-related gene (Abr). Dysregulation of Phf11 in challenged microglia and macrophages has been reported [66,67]. Among the four transcript isoforms of Phf11d detected in this study, two isoforms (Phf11d-002 and -003) were under-expressed and one isoform (Phf11d-001) was over-expressed in microglia relative to macrophages. Fig 2 depicts the relationship between the detected Phf11transcript isoforms. The predicted protein products of Phf11d-002, -003 and -001 were 337aa, 269aa, and 320aa long, respectively. Phf11 has been associated with regulation of T cell activity [68].

**Fig 3:**
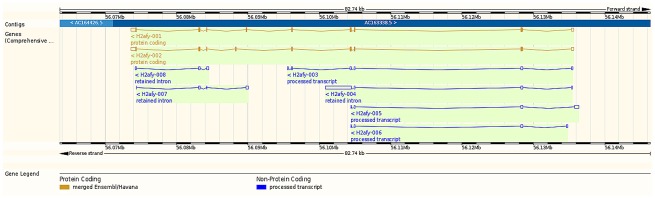
Relative location of the H2A histone family, member Y (H2afy) transcript isoforms associated with an alternative splicing event between microglia and macrophages (Ensembl, http://www.ensembl.org). Red denotes Ensembl protein coding, orange denotes merged Ensembl/Havana protein coding, and blue denotes processed transcript non-protein coding.

In the present study, isoforms H2afy-001 and -002 were under-expressed and isoforms H2afy-003 and -005 were over-expressed in microglia relative to macrophages. The proteins coded by the under-expressed isoforms H2afy-001 and -002 were the longest among those detected in this study (372aa and 369aa, respectively). Fig 3 depicts the relative location of the H2afy isoforms. Differential expression of splicing isoforms of H2afy in an oligodendrocyte lineage has been reported [69].

**Fig 4:**
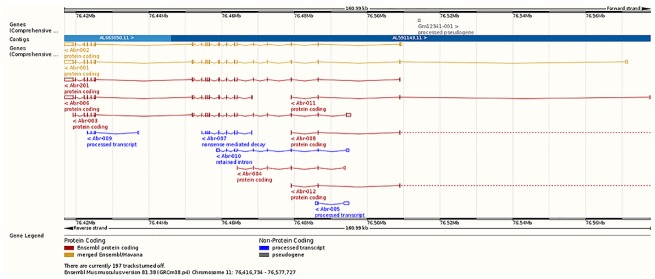
Relative location of the active BCR-related gene (Abr) transcript isoforms associated with an alternative splicing event between microglia and macrophages (Ensembl, http://www.ensembl.org). Red denotes Ensembl protein coding, orange denotes merged Ensembl/Havana protein coding, and blue denotes processed transcript non-protein coding.

Isoform Abr-201 was under-expressed whereas Abr-001 was over-expressed in microglia relative to macrophages. The products of isoforms Abr-201 and -001 are 859aa and 813aa long, respectively. Fig 4 depicts the relative location of the Abr isoforms. Absence of Abr increases the phagocytosis and chemotaxis of macrophages [70], the Abr gene products down-regulate inflammation [71], and Abr exhibits substantial genomic variability [72].

Complementary insights into the differences between microglia and macrophages were obtained from the qualitative identification of transcript isoforms detected in one cell type alone (Table 1). More genes and transcript isoforms were uniquely detected in the microglia relative to macrophages across strains. Among the genes (and transcript isoforms) detected in only one cell type, 5% to 7% of these genes (7% to 11% of the transcript isoforms) were only present in the macrophages and the rest were only present in the microglia. Functional analyses of these findings were based on 23 uniquely expressed and functionally annotated genes in the macrophage cells and 317 uniquely expressed genes in the microglia of WT mice. Three functional category clusters (Enrichment Score > 2) mainly associated with channel activity, cellular homeostasis, and cell-cell signaling were enriched among the genes detected in microglia (**Table F** in S1 File). One functional category cluster (Enrichment Score > 2) including six molecular functions related to peptidase activity, and one biological process related to proteolysis were enriched among the genes detected in macrophages. The enrichment of ion transport activity in microglia and proteolytic activity in macrophages are supported by studies of cytokine-driven autoimmune demyelinating diseases. In these cases, microglia may cause tissue injury by oxidative injury and phagocytosis meanwhile infiltrating macrophages are responsible for most of the proteolytic activity postulated to augment myelin destruction [73].

### Microglia and Macrophage Gene Networks

**Fig 5:**
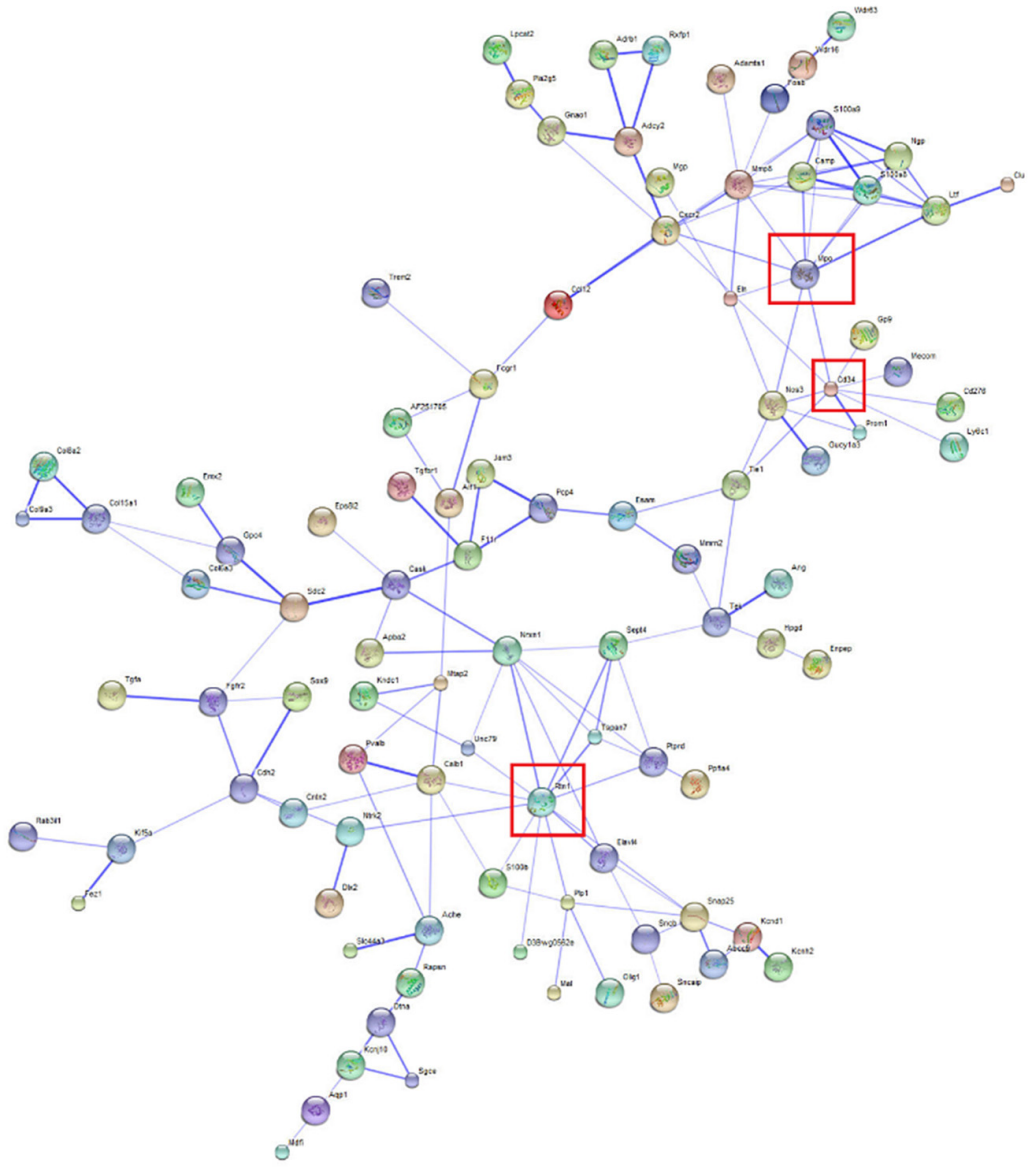
Network of genes over-expressed genes in microglia relative to macrophages in wild type mice (|log2(fold change)| > 6, FDR-adjusted P-value < 5.0 x 10^−5^) visualized using STRING. Edges represented by thicker lines denote stronger associations than thinner lines. Node size reflects the structural information associated with the protein coded by the gene. The node color facilitates visualization. Framed genes (red square) are discussed.

**Fig 6:**
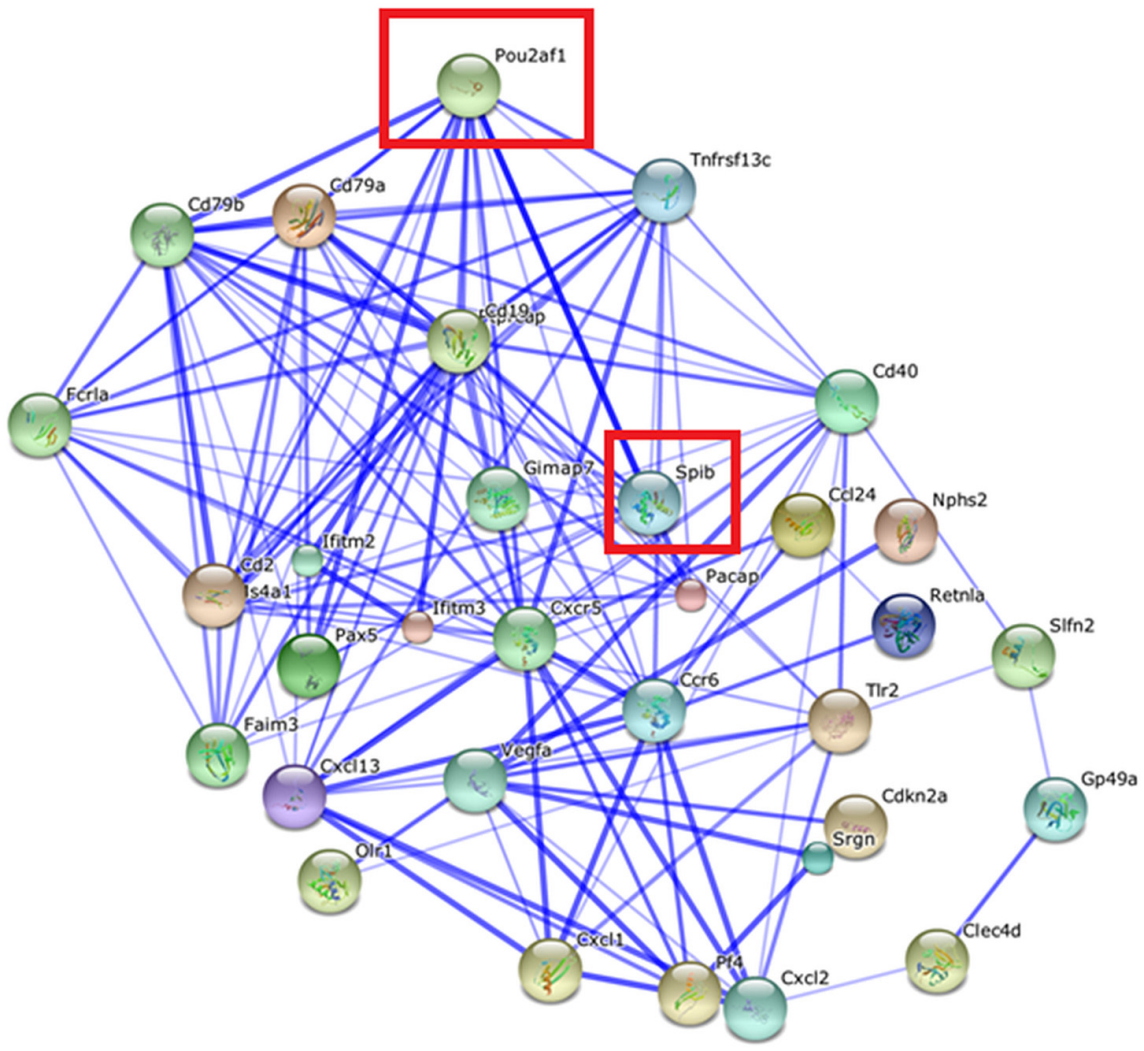
Network of genes under-expressed in microglia relative to macrophages in wild type mice (|log2(fold change)| >|6, FDR-adjusted P-value < 5.0 x 10^−5^) visualized using STRING. Edges represented by thicker lines denote stronger associations than thinner lines. Node size reflects the structural information associated with the protein coded by the gene. The node color facilitates visualization. Framed genes (red square) are discussed.

Considering the 300 most extreme differentially expressed genes between microglia and macrophages in WT (|log_2_(fold change)| > 6, FDR-adjusted P-value < 5.0 x 10^−5^), the relationships between the protein products of 99 over-expressed and 32 under-expressed genes were depicted using STRING 9.1 (Figs 5 and 6). Among them, microglia signature proteins CD34 antigen (Cd34), myeloperoxidase (Mpo), and reticulon 1 (Rtn1) showed high connectivity confirming a distinct role in microglia compared to macrophages (Fig 5) [42]. Likewise, the products of the under-expressed genes transcription factor POU domain, class 2, associating factor 1 (Pou2af1) and Spi-B transcription factor Spi-1/PU.1 related (Spib) also displayed high connectivity (Fig 6). These results suggest that changes in either one of these highly connected units can have major impact on the molecular networks.

**Fig 7:**
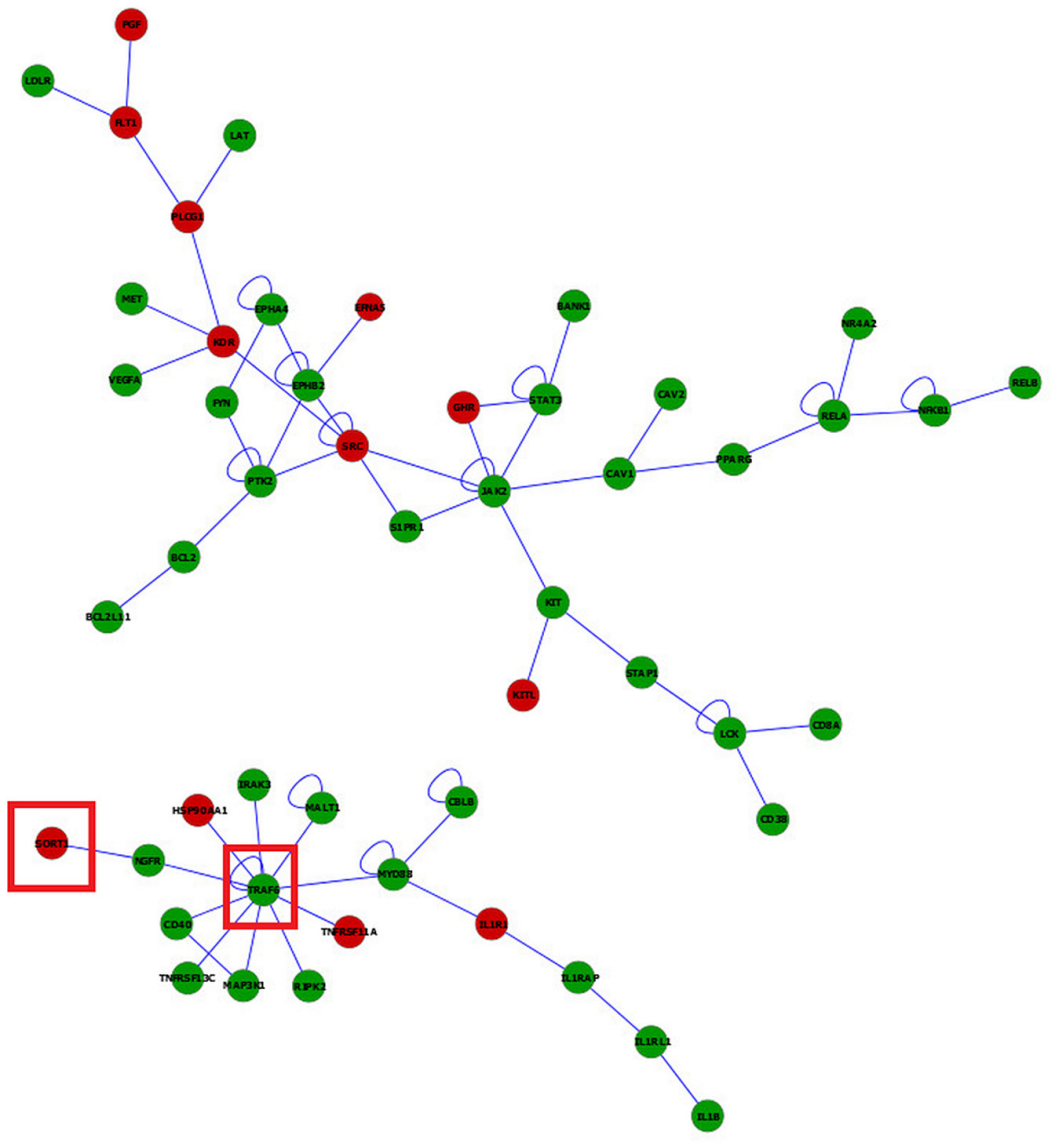
Visualization of the networks of genes differentially expressed between microglia and macrophages in wild type mice. Red (green) nodes denote genes over- (under-) expressed in microglia relative to macrophages. Node size denotes the P-value (large nodes indicate a more extreme nominal P-value < 0.0001 and small nodes indicate P-values between 0.05 and 0.0001). Edges denote known relationships between genes and framed genes (red square) are discussed.

Networks of genes differentially expressed between microglia and macrophages in the WT strain depicted using BisoGenet aided in the comparison of topologies between cell types. Informative networks including differentially expressed genes between microglia and macrophages in WT (|log_2_(fold change)| > 2, FDR-adjusted P-value < 5.0 x 10^−5^), connecting more than five genes, are presented (Fig 7). Red (green) nodes denote over- (under-) expressed in microglia relative to macrophages.

The predominance of genes under-expressed in the microglia relative to macrophages under unchallenged conditions may be a precursor for the different response to challenges of both cell types. Among the gene sub-networks, tumor necrosis factor receptor-associated factor 6 (Traf6) was connected with genes differentially expressed between cell types. Consistent with this sub-network characterization, Traf6 was affiliated to 6 of the 11 enriched categories that encompass cell activation, immune system development, apoptosis and regulation of apoptosis categories in DAVID (**Table C** in S1 File) and GSEA (**Table D** in S1 File). This sub-network also supports reports of microglia triggering cell death under potential immune challenge conditions [74].

The depicted networks aid in understanding how gene dysregulation shared by enriched functional categories can lead to major differences in the response of these cells under potential immune challenge. For example, Traf6 is crucial in interleukin-1 (IL-1) and CD40 signaling [75] and for Toll-like receptor 7 (TLR7) and 4 (TLR4) activation [76]. Traf6 was connected directly or indirectly to genes that were over-expressed in microglia relative to macrophages. Particularly, sortilin 1 (Sort1) coexists with Traf6 in the Neurotrophin signaling pathway. Sort1 is expressed in murine microglia and induces gene expression of several cytokines/chemokines including macrophage inflammatory protein (MIP)-2, monocyte chemotactic protein-1 (MCP-1), IL-1beta and tumor necrosis factor alfa (TNF-alpha).

### Transcriptomic Comparison between IDO1-KO and Wild Type Strains within Cell Type

RNA-Seq profiling confirmed the deficiency in the IDO1-KO samples of sequence reads corresponding to IDO1 and other genes affiliated to the tryptophan pathway including IDO2, tryptophan hydroxylase 2 (Tph2), indolethylamine N-methyltransferase (Inmt), acetylserotonin O-methyltransferase (Asmt), aldehyde oxidase 1, 2, and 3 (Aox1, Aox2, and Aox3). Other genes in the tryptophan pathway including tryptophan 2,3-dioxygenase (TDO), another tryptophan-degrading enzyme, were not differentially expressed between the IDO1-KO and WT strains. This is also in agreement with a previous study that compared unchallenged WT and IDO1-KO mice and reported that deletion of IDO1 did not affect the concentration of tryptophan or kynurenine in inguinal lymph nodes [77].

Relative to the comparison of cell types, fewer genes were differentially expressed between mouse strains within cell type and the majority were represented by one transcript isoform. More genes were differentially expressed between strains in macrophages (137 genes; 144 transcript isoforms) than in microglia (103 genes; 108 transcript isoforms). The limited number of genes differentially expressed between IDO1-KO and WT strains is consistent with previous reports of similar physiology, mechanical allodynia and thermal hyperalgesia between IDO1-KO and WT mice [12,78], and with normal immune system development and function reported in IDO1-KO mice [79].

Although the total number of differentially expressed genes between strains was similar across cell types, major differences in trends were observed between cell types that confirm the different role of these immune cell types. Among the genes differentially expressed between strains, 33% and 95.6% were over-expressed in IDO1-KO relative to WT mice in microglia and macrophage, respectively. Among these, eight genes differentially expressed between strains were consistent between cell types (Table 1). The list of the genes most differentially expressed (FDR-adjusted P-value < 0.001) between strains within cell type is presented in Table 6. A more extensive list of differentially expressed genes between strains is presented in **Table G** in S1 File. A list of enriched functional categories among the differentially expressed genes between strains within cell type is presented in Table 7.

**Table 6:** Differentially expressed genes (FDR-adjusted P-value < 0.001) between wild type (WT) and IDO1-knockout (IDO1-KO) mice within cell type, including log_2_(fold ratio) and supporting literature review reference.

Gene Symbol	Gene Name	Log_2_(WT/ IDO1-KO)	Literature Review References
**Microglia**			
Bcas1	breast carcinoma amplified sequence 1	2.32	[80]
Kif5a	kinesin family member 5A	2.25	[81]
Mbp	myelin basic protein	2.11	[82]
Mobp	myelin-associated oligodendrocytic basic protein	1.87	[83]
Mid1	midline 1	1.61	[84]
Plekhb1	pleckstrin homology domain containing, family B (evectins) member 1	1.34	[85]
Kif5c	kinesin family member 5C	1.17	[86]
S100b	S100 protein, beta polypeptide, neural	1.09	[87]
Olig1	oligodendrocyte transcription factor 1	1.04	[88]
Stmn4	stathmin-like 4	1.04	[89]
Mog	myelin oligodendrocyte glycoprotein	1.03	[88]
Tubb4a	tubulin, beta 4A class IVA	0.97	[88]
Cldn11	claudin 11	0.95	[88]
Plp1	proteolipid protein (myelin) 1	0.94	[88]
Tmem88b	transmembrane protein 88B	0.93	[88]
Gpr37	G protein-coupled receptor 37	0.93	[88]
Il33	interleukin 33	0.87	[7]
Cntn2	contactin 2	0.85	[88]
Efnb3	ephrin B3	0.83	[88]
Pllp	plasma membrane proteolipid	0.80	[88]
Slc15a2	solute carrier family 15 (H+/peptide transporter), member 2	0.71	[57]
1810011O10Rik	RIKEN cDNA 1810011O10 gene	0.66	[57]
Irg1, Cad	immunoresponsive gene 1	-0.94	[57]
Gbp2	guanylate binding protein 2	-0.98	[90]
Oas3	2'-5' oligoadenylate synthetase 3	-1.14	[90]
Asprv1	aspartic peptidase, retroviral-like 1	-1.43	[91]
Cfb	complement factor B	-1.44	[57]
Lrg1	leucine-rich alpha-2-glycoprotein 1	-1.45	[88]
Plac8	placenta-specific 8	-1.58	[92]
Ifitm1	interferon induced transmembrane protein 1	-1.61	[7]
Pomc	pro-opiomelanocortin-alpha	-1.88	[57]
Steap4	STEAP family member 4	-2.28	[57]
Saa3	serum amyloid A 3	-3.32	[90]
Darc	atypical chemokine receptor 1 (Duffy blood group)	-4.21	[88]
**Macrophages**			
Slc15a2	solute carrier family 15 (H+/peptide transporter), member 2	1.02	[93]
Rpl39	ribosomal protein L39	0.68	[92]
Arg1	arginase, liver	-0.65	[94]
Ckap2l	cytoskeleton associated protein 2-like	-0.78	[92]
Sema3c	sema domain, immunoglobulin domain (Ig), short basic domain, secreted, (semaphorin) 3C	-0.94	[92]
Pdpn	podoplanin	-0.97	[92]
Ifi205	interferon activated gene 205	-0.97	[90]
Phlda1	pleckstrin homology-like domain, family A, member 1	-0.99	[7]
Ccl2	chemokine (C-C motif) ligand 2	-1.03	[7]
Ch25h	cholesterol 25-hydroxylase	-1.11	[95]
Edn1	endothelin 1	-1.12	[92]
Prok2	prokineticin 2	-1.18	[93]
Tnfsf14	tumor necrosis factor (ligand) superfamily, member 14	-1.22	[93]
F3	coagulation factor III	-1.25	[90]
Slamf1	signaling lymphocytic activation molecule family member 1	-1.33	[7]
Steap4	STEAP family member 4	-1.34	[93]
Ccl5	chemokine (C-C motif) ligand 5	-1.45	[91]
Hba-a2	hemoglobin alpha, adult chain 2	-1.48	[91]
Ccrn4l	CCR4 carbon catabolite repression 4-like	-1.49	[92]
Il10	interleukin 10	-1.52	[7]
Hbegf	heparin-binding EGF-like growth factor	-1.52	[93]
Lipg	lipase, endothelial	-1.65	[92]
Ifih1	interferon induced with helicase C domain 1	-1.66	[92]
Rsad2	radical S-adenosyl methionine domain containing 2	-1.74	[92]
Ccl7	chemokine (C-C motif) ligand 7	-1.76	[90]
Tulp4	tubby like protein 4	-1.76	[93]
Isg15	ISG15 ubiquitin-like modifier	-1.92	[92]
Csf2	colony stimulating factor 2 (granulocyte-macrophage)	-2.46	[96]
Oasl1	2'-5' oligoadenylate synthetase-like 1	-2.74	[90]
Sele	selectin, endothelial cell	-3.04	[91]
Csf3	colony stimulating factor 3 (granulocyte)	-3.53	[7]

**Table 7:** Enriched (DAVID Enrichment score ES > 2) functional cluster of categories encompassing transcript isoforms differentially expressed between IDO1-knockout and wild type mice within cell type.

Cell Type and Category^1^	Terms^2^	Gene Count	P-value	ES
**Microglia**				
Cluster 1				2.61
GO_BP_FAT	GO:0007272:Ensheathment of neurons	5	4.64E-05	
GO_BP_FAT	GO:0008366: Axon ensheathment	5	4.64E-05	
GO_BP_FAT	GO:0019228:Regulation of action potential in neuron	5	8.20E-05	
GO_BP_FAT	GO:0001508:Regulation of action potential	5	1.68E-04	
GO_BP_FAT	GO:0042391:Regulation of membrane potential	6	3.64E-04	
GO_BP_FAT	GO:0042552:Myelination	4	8.97E-04	
GO_BP_FAT	GO:0006873:Cellular ion homeostasis	7	2.22E-03	
GO_BP_FAT	GO:0055082:Cellular chemical homeostasis	7	2.54E-03	
GO_BP_FAT	GO:0050801:Ion homeostasis	7	3.94E-03	
GO_BP_FAT	GO:0019226:Transmission of nerve impulse	6	6.13E-03	
GO_BP_FAT	GO:0019725:Cellular homeostasis	7	8.37E-03	
GO_BP_FAT	GO:0048878:Chemical homeostasis	7	1.12E-02	
GO_BP_FAT	GO:0042592:Homeostatic process	8	3.03E-02	
GOTERM_MF_FAT	GO:0005198:Structural molecule activity	5	1.91E-01	
GOTERM_BP_FAT	GO:0050877:Neurological system process	7	8.79E-01	
**Macrophages**				
Cluster 1				8.39
GOTERM_MF_FAT	GO:0005125:Cytokine activity	18	3.16E-15	
KEGG_PATHWAY	mmu04060:Cytokine-cytokine receptor interaction	18	1.57E-10	
GO_BP_FAT	GO:0006955:Immune response	19	8.38E-09	
KEGG_PATHWAY	mmu04630:Jak-STAT signaling pathway	11	2.61E-06	
GOTERM_BP_FAT	GO:0006952:Defense response	13	1.08E-04	
Cluster 2				3.35
GOTERM_MF_FAT	GO:0008009:Chemokine activity	6	5.36E-06	
GOTERM_MF_FAT	GO:0042379:Chemokine receptor binding	6	6.12E-06	
GO_BP_FAT	GO:0009611:Response to wounding	13	8.89E-06	
GO_BP_FAT	GO:0006952:Defense response	13	1.08E-04	
GO_BP_FAT	GO:0006935:Chemotaxis	7	1.50E-04	
GO_BP_FAT	GO:0042330:Taxis	7	1.50E-04	
GO_BP_FAT	GO:0006954:Inflammatory response	8	1.35E-03	
KEGG_PATHWAY	mmu04621:NOD-like receptor signaling pathway	5	3.61E-03	
GO_BP_FAT	GO:0007626:Locomotory behavior	7	8.39E-03	
GO_BP_FAT	GO:0007610:Behavior	9	9.96E-03	
KEGG_PATHWAY	mmu04062:Chemokine signaling pathway	7	1.05E-02	
KEGG_PATHWAY	mmu04620:Toll-like receptor signaling pathway	5	1.84E-02	
Cluster 3				2.40
GO_BP_FAT	GO:0001944:Vasculature development	9	5.08E-04	
GO_BP_FAT	GO:0001568:Blood vessel development	8	2.14E-03	
GO_BP_FAT	GO:0048514:Blood vessel morphogenesis	7	3.40E-03	
GO_BP_FAT	GO:0044057:Regulation of system process	6	1.64E-02	
GO_BP_FAT	GO:0001525Angiogenesis	5	1.67E-02	
Cluster 4				2.24
GO_BP_FAT	GO:0030335:Positive regulation of cell migration	5	1.51E-04	
GO_BP_FAT	GO:0051272:Positive regulation of cell motion	5	2.27E-04	
GO_BP_FAT	GO:0040017:Positive regulation of locomotion	5	2.99E-04	
GO_BP_FAT	GO:0030334:Regulation of cell migration	5	4.70E-03	
GO_BP_FAT	GO:0051270:Regulation of cell motion	5	7.99E-03	
GO_BP_FAT	GO:0040012:Regulation of locomotion	5	8.79E-03	
GO_BP_FAT	GO:0007155:Cell adhesion	5	5.97E-01	
GO_BP_FAT	GO:0022610:Biological adhesion	5	5.98E-01	

^1^ Each row corresponds to a Functional Annotation Tool (FAT) GO category inside a cluster.

^2^ GO terms inside each cluster.

An interesting finding among the differentially expressed genes between strains was the over-expression of cholesterol 25-Hydroxylase (Ch25h) in IDO1-KO relative to WT macrophages. Ch25h plays an important role in regulating lipid metabolism, gene expression, and immune activation [97]. Ch25h is affiliated to the enriched GO categories fatty acid metabolic process and transition metal ion binding function albeit below the threshold established for significant enrichment. This profile could be an important compensatory mechanism that regulates the immune system in IDO1-KO mice.

Another two genes differentially over-expressed in IDO1-KO macrophages relative to WT macrophages were colony stimulating factor 2 granulocyte-macrophage (Csf2) and colony stimulating factor 3 granulocyte (Csf3). Csf2 and Cfs3 were affiliated to four functional categories within the most significant enriched functional cluster 1 (Table 7). Csf2 and Csf3 support the development of macrophages, and lymphocytes, induce the expression of pro-inflammatory cytokines and Janus kinase-signal transducer and activator transcription (Jak-STAT) signaling pathway [98]. In microglia, serum amyloid A 3 (Saa3) and immunoresponsive gene 1 (Irg1) were over-expressed in IDO1-KO relative to WT. Saa3 is expressed in immune cells [99] and Irg1 links the metabolic and immune systems [100]. These genes are also associated to immune response and could be a compensatory immunomodulation pathway in IDO1-KO mice. Like Ch25h, Saa3 and Irg1 are affiliated to functional categories that did not surpass the enrichment threshold.

Breast carcinoma amplified sequence 1 (Bcas1) and SRY (sex determining region Y)-box 10 (Sox10) were under-expressed in IDO1-KO relative to WT. Bcas1 was affiliated to the functional categories of protein binding and protein dimerization activity and has been associated with Parkinson’s disease and schizophrenia [101,102]. Sox10 was affiliated to the functional categories of transcription factor activity and regulation and is a transcription factor that encompasses mutations associated with neurological phenotypes such as a complex neurocristopathy and Waardenburg-Shah syndrome [53]. These findings offer insights into the role of IDO1 in the transcriptome profile that could be linked to neurological disorders.

### Functional Analysis of Differences between IDO1-KO and Wild Type Strains

Functional categories enriched among the genes differentially expressed between strains included cytokine interaction, cytokine activity, chemokines activity, cell migration, and cell adhesion (Table 7). The genes associated with the immunological response category were over-expressed in IDO1-KO relative to WT indicating a lack of immunosuppressive response in IDO1-KO. These findings suggest that the balance between response and tolerance mediated by the immunosuppressive activity of IDO1 in unchallenged conditions may be altered due to the depletion of this enzyme in IDO1-KO mice.

Biological processes enriched among the genes differentially expressed between IDO1-KO and WT in the microglia included ensheathment (envelop) of neurons and the cellular, chemical, and ion homeostasis, and homeostatic process (Table 7). Consistent with our findings, microglia promotes neuronal growth and survival [103] and maintains homeostasis in the brain [104]. Proteolipid protein (myelin) 1 (PLP1) and gap junction protein, gamma 3 (GJC3) were affiliated to the categories ensheathment of neurons and were under-expressed in IDO1-KO relative to WT mice. Reduction in levels of PLP1 products affects myelin stability and axonal integrity and mutations in PLP1 have been associated with neurological disorders [105]. GJC3 is over-expressed in microglia in the area subjacent to trauma and is up-regulated in glioma-associated microglia/ macrophages [106]. The results of this functional analysis highlight the role of IDO1 in the transcriptome profile under unchallenged conditions.

The genes differentially expressed between WT and IDO1-KO mice detected in this study were in agreement with comparative studies of these strains. Similarly, enriched functional categories detected in this study are consistent with the reported response of IDO1-KO mice to infection [107]. Loss of IDO activity (such as that in IDO1-KO mice) has been associated with reduced morbidity during influenza infection and accelerated recovery after viral clearance; meanwhile IDO induction restrained and shaped host T cell responses [107]. Our results confirmed that IDO-mediating immune suppression and tolerance are important negative regulatory mechanisms under unchallenged conditions [108]. In a model of multiple sclerosis, IDO deficiency enhanced encephalitogenic T cell responses, reduced regulatory T cell (Treg), and exacerbated experimental autoimmune encephalomyelitis [109]. In cancer research, tumors cells usually over-expressed IDO, reinforcing a cycle of antigen tolerance leaning the immune system to change from hostile to less reactive with the tumor cells and therefore, IDO suppression cooperates with chemotherapy [110,111].

The enriched functional categories among genes differentially expressed between strains under unchallenged conditions are consistent with published results under challenged conditions and could relate to the capability of these strains to respond to potential challenge. The higher number of differentially expressed genes between strains with and without IDO1 (WT and IDO1-KO, respectively) in macrophages relative to microglia and enriched categories including cytokine and chemokine activities (Table 7) are consistent with reports that elevated lung IDO1 activity was associated with suppressed lung Th17 response and modified lung pathology mediated by IL-17 [107]. Furthermore, maintained kynurenine release associated with higher IDO1 activity could lower blood pressure and influence ‘sickness induced depression’ behaviors [107,112]. This link is consistent with our finding of enriched vasculature development category among genes differentially expressed between WT and IDO1-KO in macrophages (Table 7).

### IDO1-KO and Wild Type Gene Networks

**Fig 8:**
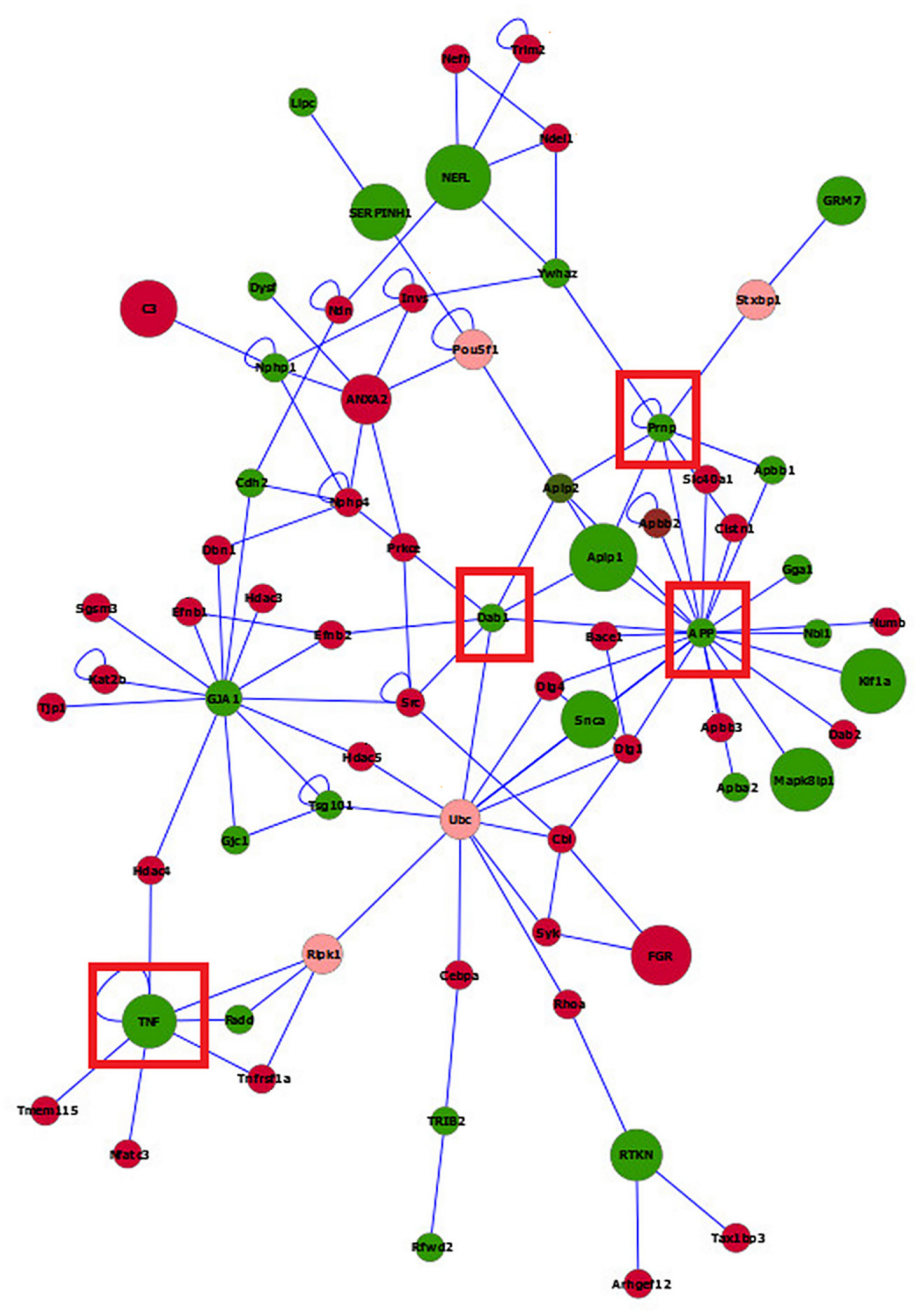
Visualization of the networks of genes differentially expressed between IDO1-knock out (IDO1-KO) and wild type (WT) mice in microglia. Red (green) nodes indicate genes over- (under-) expressed in IDO1-KO relative to wild type. Pink nodes denote a maximum of two intermediate genes between target genes. Node size denotes the P-value (large nodes indicate a more extreme nominal P-value < 0.0001 and small nodes indicate P-value between 0.05 and 0.0001). Edges denote known relationships between genes and framed genes (red square) are discussed.

**Fig 9:**
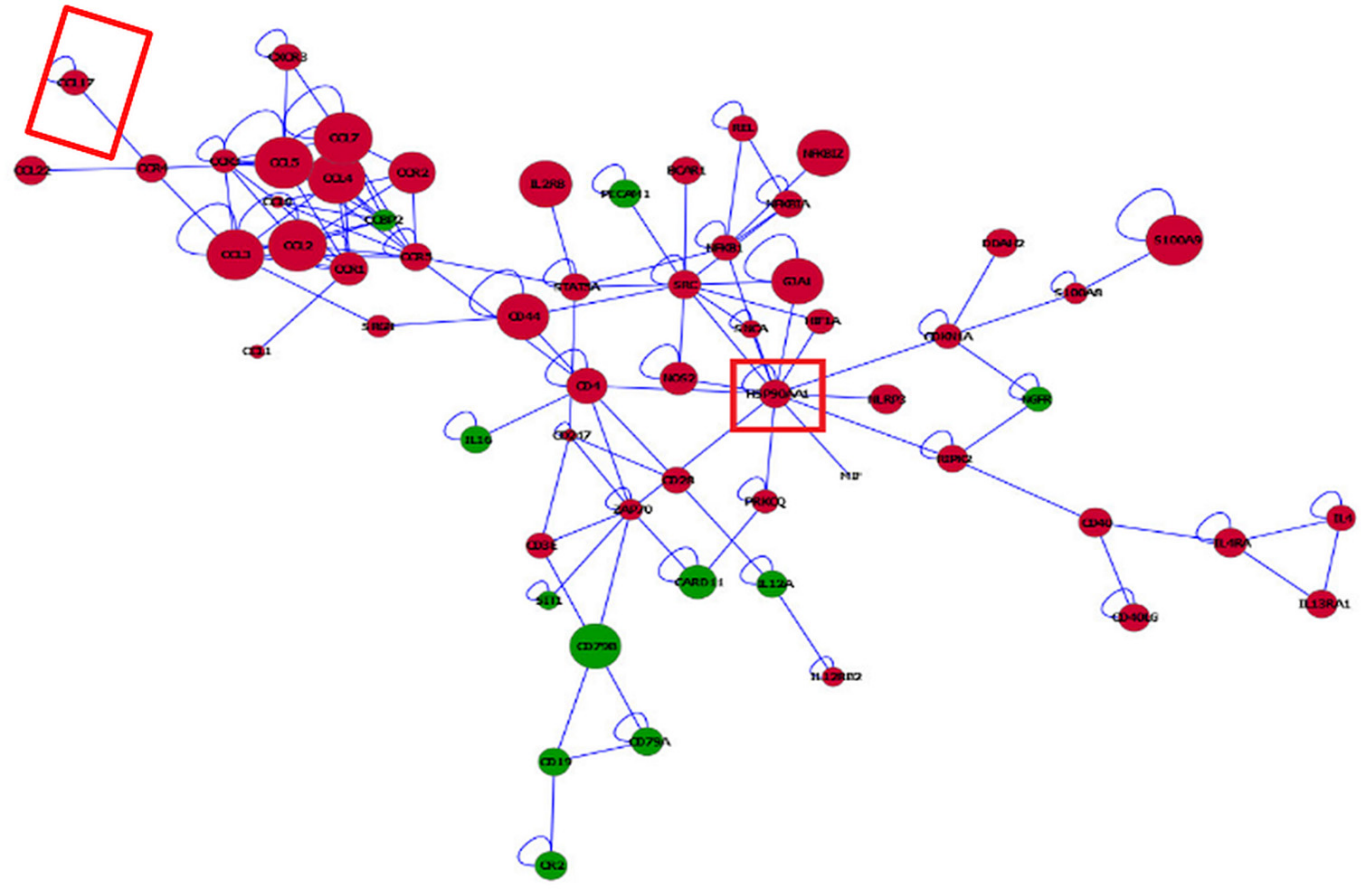
Visualization of the networks of genes differentially expressed between IDO1-knock out (IDO1-KO) and wild type (WT) mice in macrophages. Red (green) nodes indicate genes over- (under-) expressed in IDO1-KO relative to wild type. Node size indicate the P-value (large nodes indicate a more extreme nominal P-value < 0.0001 and small nodes indicate a P-value between 0.05 and 0.0001). Edges denote known relationships between genes and framed genes (red square) are discussed.

Insights into differences between IDO1-KO and WT in both microglia (Fig 8) and macrophages (Fig 9) were gained from the visualization of the network of genes differentially expressed using BisoGenet. To facilitate the visualization of gene connectivity, the networks include genes that had a differential expression nominal P-value < 0.05 although the differential expression of most genes surpassed the previously established significance threshold. Networks including at least five connected genes are presented to assist with interpretation. The microglia network included 70 gene nodes and 53% of the genes were over-expressed in IDO1-KO relative to WT mice (Fig 8). The macrophage network included 72 gene nodes, and the 75% of the nodes were over-expressed in IDO1-KO relative to WT mice (Fig 9). Meanwhile the number of over- and under-expressed genes between strains was similar in the microglia network, the number of genes over-expressed in IDO1-KO relative to WT mice was higher in the macrophage network. This result suggests a more consistent directionality of the impact of the IDO1-KO in the macrophage transcriptome that is less systemic in the microglia.

Network reconstruction demonstrated that meanwhile the highly differentially expressed genes between strains were well-distributed in the microglia network, the most differentially expressed genes were concentrated in a C-C module in the macrophage network. In the macrophage network, chemokine (C-C motif) ligand 3 (Ccl3), chemokine (C-C motif) ligand 7 (Ccl7), chemokine (C-C motif) ligand 5 (Ccl5), chemokine (C-C motif) ligand 4 (Ccl4), and chemokine (C-C motif) ligand 2 (Ccl2) were over-expressed in IDO1-KO relative to WT. These genes were annotated to a number of categories related with cytokine and chemokine activity presented in the enriched functional cluster 1 and 2 in Table 7. These findings are consistent with reports that microglia activation is preceded by increased gene expression in the immune modulatory chemokine chemokine (C-C motif) ligand 4 [113].

In the microglia network, App constituted the most connected gene node, linked to 21 other genes. App was affiliated to a number of enriched categories related with homeostasis within one functional cluster in microglia (Table 7) and was under-expressed in IDO1-KO relative to WT. Another highly connected gene was prion protein (Prnp) that was linked to eight other genes and was also under-expressed in IDO1-KO relative to WT. This gene was affiliated to at least three enriched categories related to ion and chemical homeostasis (Table 7). Disabled 1 (Dab1) and tumor necrosis factor (TNF) were also under-expressed in IDO1-KO relative to WT and were connected to seven other genes, although the affiliated functional categories were enriched below the threshold.

In the macrophage network, heat shock protein 90, alpha (cytosolic), class A member 1 (Hsp90aa1) was the most connected gene (linked to 14 genes) in the network. This gene was over-expressed in the IDO1-KO relative to WT and our results are consistent with macrophages studies indicating that heat stress proteins are associated with the development of innate and adaptive immune responses [114].

## Conclusions

The similarity in transcriptome profiles between strains confirmed that IDO1-KO transcriptome is not pervasively dysregulated by depletion of IDO1 under unchallenged conditions. However, the depletion of IDO1 could alter the balance between defense and tolerance or between M1 and M2 microglia phenotypes. This dysregulation could result in the over-expression of genes that link metabolism and immune response as a compensatory mechanism to regulate the immune system under potential challenge conditions. Our results also highlight the large number of differentially expressed genes between microglia and macrophages within and between strains under unchallenged conditions. Network analysis depicted a predominance of genes under-expressed in microglia relative to macrophages that could be a precursor of the differential response of these immune cells to challenge. Our study of transcript isoforms and alternative splicing events further augmented our understanding of the role of IDO1 in the microglia transcriptome under unchallenged conditions.

## Supporting Information

S1 File Supplementary results. Table A. Average number of sequence reads available; percentage of reads mapped (RM), percentage of reads uniquely mapped (UM); percentage of reads mapped to multiple locations (MM); and concordant pair mapping percentage (CPM) to the mouse genome by strain, and cell type group. Table B. Differentially expressed genes (FDR-adjusted P-value < 5.0 x 10^−5^) between microglia and macrophages in wild type mice, including log_2_(fold ratio) and supporting literature review reference. Table C. Enriched (DAVID Enrichment score ES > 2) functional clusters of categories encompassing differentially expressed transcript isoforms between microglia and macrophages in wild type mice. Table D. Gene Set Enrichment Analysis categories among transcript isoforms under-expressed (FDR-adjusted P-value < 0.05 with > 10 transcript isoforms) and over- expressed (P-value < 0.01 and > 10 transcript isoforms) in microglia relative to macrophages in wild type mice. Table E. Genes exhibiting an alternative splicing events between microglia and macrophages in wild type mice including at least two transcript isoforms and at least one over- or under-expressed (FDR-adjusted P-value < 0.05) transcript isoform between cell types. Table F. Enriched (DAVID Enrichment score ES > 2) functional cluster of categories encompassing transcript isoforms expressed solely in macrophages and in microglia in wild type mice. Table G. Differentially expressed genes (FDR-adjusted P-value < 0.05) between wild type (WT) and IDO1-knockout (KO) mice within cell type, including log_2_(fold ratio) and supporting literature review reference. Fig A. Distribution of expression levels expressed in fragments per kilobase of transcript per million mapped fragments (FPKMs) for 28,403 annotated genes with at least five reads per transcript in IDO1-knockout microglia (IDO1_KO_Brain), wild type microglia (Wild_Brain), IDO1-knockout macrophages (IDO1_KO_Peritoneo), and wild type macrophages (Wild_Peritoneo).
(DOCX)
