# Supplementary material for: Differential Transcriptome Networks between IDO1-Knockout and Wild-Type Mice in Brain Microglia and Macrophages
Source: PLoS One. 2016 Jun 17;11(6):e0157727. doi: 10.1371/journal.pone.0157727 (PMC4912085; doi:10.1371/journal.pone.0157727)
Supplement: S1 File — Table A. Average number of sequence reads available; percentage of reads mapped (RM), percentage of reads uniquely mapped (UM); percentage of reads mapped to multiple locations (MM); and concordant pair mapping percentage (CPM) to the mouse genome by strain, and cell type group. Table B. Differentially expressed genes (FDR-adjusted P-value < 5.0 x 10^−5^) between microglia and macrophages in wild type mice, including log2(fold ratio) and supporting literature review reference. Table C. Enriched (DAVID Enrichment score ES > 2) functional clusters of categories encompassing differentially expressed transcript isoforms between microglia and macrophages in wild type mice. Table D. Gene Set Enrichment Analysis categories among transcript isoforms under-expressed (FDR-adjusted P-value < 0.05 with > 10 transcript isoforms) and over- expressed (P-value < 0.01 and > 10 transcript isoforms) in microglia relative to macrophages in wild type mice. Table E. Genes exhibiting an alternative splicing events between microglia and macrophages in wild type mice including at least two transcript isoforms and at least one over- or under-expressed (FDR-adjusted P-value < 0.05) transcript isoform between cell types. Table F. Enriched (DAVID Enrichment score ES > 2) functional cluster of categories encompassing transcript isoforms expressed solely in macrophages and in microglia in wild type mice. Table G. Differentially expressed genes (FDR-adjusted P-value < 0.05) between wild type (WT) and IDO1-knockout (KO) mice within cell type, including log2(fold ratio) and supporting literature review reference. Fig A. Distribution of expression levels expressed in fragments per kilobase of transcript per million mapped fragments (FPKMs) for 28,403 annotated genes with at least five reads per transcript in IDO1-knockout microglia (IDO1_KO_Brain), wild type microglia (Wild_Brain), IDO1-knockout macrophages (IDO1_KO_Peritoneo), and wild type macrophages (Wild_Peritoneo). (DOCX) [file pone.0157727.s001.docx]

Table A. Average number of sequence reads available, percentage of reads mapped (RM); percentage of reads uniquely mapped (UM); percentage of reads mapped to multiple locations (MM); concordant pair mapping percentage (CPM) to the mouse genome by cell type and strain.

| Sample^1^ | Total reads | RM (%) | UM (%) | MM (%) | CPM (%) |
| --- | --- | --- | --- | --- | --- |
| Microglia IDO1-KO | 65,528,979 | 88.46 | 76.34 | 13.70 | 83.94 |
| Microglia WT | 57,432,778 | 88.15 | 75.87 | 13.94 | 83.63 |
| Macrophage IDO1-KO | 62,929,589 | 92.15 | 75.27 | 18.31 | 86.25 |
| Macrophage WT | 55,243,410 | 92.06 | 74.87 | 18.67 | 86.29 |

^1^Sample nomenclature: cell type: microglia or macrophage and strain: IDO1-knockout (IDO1-KO) and wild type (WT)

Table B. Most extreme differentially expressed genes (FDR-adjusted P-value < 5.0 x 10^-5^) between microglia and macrophages in wild type mice, including log_2_(fold ratio).

| Gene Symbol | Gene name | Log_2_(Macrophage/Microglia) |
| --- | --- | --- |
| Slc2a5 | Solute carrier family 2 (facilitated glucose transporter), member 5 | -12.950 |
| Gpr56 | G protein-coupled receptor 56 | -12.499 |
| Olfml3 | Olfactomedin-like 3 | -12.452 |
| Siglech | Sialic acid binding Ig-like lectin H | -11.639 |
| Crybb1 | Crystallin, beta B1 | -10.468 |
| S100a9 | S100 calcium binding protein A9 (calgranulin B) | -10.017 |
| Cask | Calcium/calmodulin-dependent serine protein kinase | -9.766 |
| Serpine2 | Serine (or cysteine) peptidase inhibitor, clade E, member 2 | -8.602 |
| Ang | Angiogenin, ribonuclease, RNase A family, 5 | -8.369 |
| Hpgd | Hydroxyprostaglandin dehydrogenase 15 (NAD) | -7.650 |
| Rab3il1 | RAB3A interacting protein (rabin3)-like 1 | -7.492 |
| Fosb | FBJ osteosarcoma oncogene B | -7.308 |
| Lag3 | Lymphocyte-activation gene 3 | -7.249 |
| S100a8 | S100 calcium binding protein A8 (calgranulin A) | -7.203 |
| F11r | F11 receptor | -6.948 |
| Fscn1 | Fascin homolog 1, actin bundling protein | -6.157 |
| St3gal6 | ST3 beta-galactoside alpha-2,3-sialyltransferase 6 | -5.679 |
| Phyhd1 | Phytanoyl-CoA dioxygenase domain containing 1 | -5.657 |
| Trem2 | Triggering receptor expressed on myeloid cells 2 | -5.494 |
| Aif1 | Allograft inflammatory factor 1 | -5.297 |
| Golm1 | Golgi membrane protein 1 | -4.717 |
| AF251705 | cDNA sequence AF251705 | -4.608 |
| Lpcat2 | Lysophosphatidylcholine acyltransferase 2 | -4.031 |
| Ckb | Creatine kinase, brain | -3.754 |
| Srgap2 | SLIT-ROBO Rho GTPase activating protein 2 | -3.667 |
| R74862 | Expressed sequence R74862 | -3.481 |
| Tmem173 | Transmembrane protein 173 | -3.205 |
| Tgfbr1 | Transforming growth factor, beta receptor I | -3.085 |
| Abhd12 | Abhydrolase domain containing 12 | -3.066 |
| Tagln2 | Transgelin 2 | 3.080 |
| Tlr2 | Toll-like receptor 2 | 3.225 |
| Ifitm3 | Interferon induced transmembrane protein 3 | 3.274 |
| Srgn | Serglycin | 3.275 |
| Ninj1 | Ninjurin 1 | 3.439 |
| Capg | Capping protein (actin filament), gelsolin-like | 3.579 |
| Crip1 | Cysteine-rich protein 1 (intestinal) | 3.631 |
| Smpdl3a | Sphingomyelin phosphodiesterase, acid-like 3A | 3.795 |
| Nupr1 | Nuclear protein transcription regulator 1 | 3.980 |
| Ifitm2 | Interferon induced transmembrane protein 2 | 4.043 |
| Fxyd5 | FXYD domain-containing ion transport regulator 5 | 4.093 |
| Slfn2 | Schlafen 2 | 4.130 |
| S100a4 | S100 calcium binding protein A4 | 4.260 |
| Plac8 | Placenta-specific 8 | 4.368 |
| Cd79b | CD79B antigen | 4.867 |
| Pf4 | Platelet factor 4 | 5.106 |
| Lilrb4 | Leukocyte immunoglobulin-like receptor, subfamily B, member 4 | 5.141 |
| Tnfaip2 | Tumor necrosis factor, alpha-induced protein 2 | 5.329 |
| Cxcl2 | Chemokine (C-X-C motif) ligand 2 | 5.882 |
| Wfdc17 | WAP four-disulfide core domain 17 | 6.375 |
| Cxcl1 | Chemokine (C-X-C motif) ligand 1 | 7.333 |
| Alox15 | Arachidonate 15-lipoxygenase | 8.052 |
| Ccl24 | Chemokine (C-C motif) ligand 24 | 8.092 |
| 4921530D09Rik | Coiled-coil domain containing 183 | 8.144 |
| Clec4d | C-type lectin domain family 4, member d | 8.447 |
| Retnla | Resistin like alpha | 9.290 |
| Olr1 | Oxidized low density lipoprotein (lectin-like) receptor 1 | 10.002 |
| Lyz1 | Lysozyme 1 | 10.887 |
| Cd5l | CD5 antigen-like | 11.130 |
| Cxcl13 | Chemokine (C-X-C motif) ligand 13 | 11.746 |

Table C. Enriched (DAVID Enrichment score ES > 2) functional clusters of categories encompassing differentially expressed transcript isoforms between microglia and macrophages in wild type mice.

| Cluster and Category^1^ | Terms^2^ | Gene Count | P-value | **ES** |
| --- | --- | --- | --- | --- |
| **Cluster 1** |  |  |  | **11.85** |
| GO_BP_FAT | GO:0007155:cell adhesion | 146 | 7.17E-17 |  |
| GO_BP_FAT | GO:0022610:biological adhesion | 146 | 1.96E-16 |  |
| GO_BP_FAT | GO:0016337:cell-cell adhesion | 52 | 1.99E-04 |  |
| **Cluster 2** |  |  |  | **8.07** |
| GO_ MF_FAT | GO:0030246:carbohydrate binding | 89 | 4.67E-13 |  |
| GO_ MF_FAT | GO:0001871:pattern binding | 42 | 1.03E-08 |  |
| GO_ MF_FAT | GO:0030247:polysaccharide binding | 42 | 1.03E-08 |  |
| GO_ MF_FAT | GO:0005539:glycosaminoglycan binding | 36 | 3.86E-07 |  |
| GO_ MF_FAT | GO:0008201:heparin binding | 28 | 2.35E-06 |  |
| **Cluster 3** |  |  |  | **6.67** |
| GO_BP_FAT | GO:0009611:response to wounding | 85 | 1.12E-08 |  |
| GO_BP_FAT | GO:0006954:inflammatory response | 62 | 1.48E-08 |  |
| GO_BP_FAT | GO:0006952:defense response | 89 | 5.76E-05 |  |
| **Cluster 4** |  |  |  | **5.93** |
| GO_BP_FAT | GO:0006928:cell motion | 86 | 7.84E-08 |  |
| GO_BP_FAT | GO:0030030:cell projection organization | 77 | 1.14E-07 |  |
| GO_BP_FAT | GO:0031175:neuron projection development | 58 | 1.69E-07 |  |
| GO_BP_FAT | GO:0048812:neuron projection morphogenesis | 49 | 4.13E-07 |  |
| GO_BP_FAT | GO:0007411:axon guidance | 33 | 4.86E-07 |  |
| GO_BP_FAT | GO:0000902:cell morphogenesis | 73 | 6.05E-07 |  |
| GO_BP_FAT | GO:0007409:axonogenesis | 46 | 6.49E-07 |  |
| GO_BP_FAT | GO:0048858:cell projection morphogenesis | 53 | 9.74E-07 |  |
| GO_BP_FAT | GO:0000904:cell morphogenesis involved in differentiation | 54 | 2.02E-06 |  |
| GO_BP_FAT | GO:0032990:cell part morphogenesis | 53 | 4.57E-06 |  |
| GO_BP_FAT | GO:0032989:cellular component morphogenesis | 77 | 5.73E-06 |  |
| GO_BP_FAT | GO:0048667:cell morphogenesis involved in neuron differentiation | 47 | 6.86E-06 |  |
| GO_BP_FAT | GO:0030182:neuron differentiation | 84 | 1.09E-05 |  |
| GO_BP_FAT | GO:0048666:neuron development | 65 | 2.07E-05 |  |
| **Cluster 5** |  |  |  | **5.08** |
| GO_BP_FAT | GO:0006928:cell motion | 86 | 7.84E-08 |  |
| GO_BP_FAT | GO:0016477:cell migration | 58 | 4.74E-06 |  |
| GO_BP_FAT | GO:0051674:localization of cell | 61 | 1.12E-04 |  |
| GO_BP_FAT | GO:0048870:cell motility | 61 | 1.12E-04 |  |
| **Cluster 6** |  |  |  | **4.60** |
| GO_ MF_FAT | GO:0030695:GTPase regulator activity | 86 | 9.56E-09 |  |
| GO_ MF_FAT | GO:0060589:nucleoside-triphosphatase regulator activity | 86 | 2.15E-08 |  |
| GO_BP_FAT | GO:0051056:regulation of small GTPase mediated signal transduction | 58 | 8.39E-07 |  |
| GO_ MF_FAT | GO:0005085:guanyl-nucleotide exchange factor activity | 38 | 3.13E-05 |  |
| GO_ MF_FAT | GO:0005083:small GTPase regulator activity | 51 | 4.22E-05 |  |
| GO_ MF_FAT | GO:0008047:enzyme activator activity | 54 | 1.09E-04 |  |
| GO_ MF_FAT | GO:0005088:Ras guanyl-nucleotide exchange factor activity | 24 | 2.10E-04 |  |
| GO_ MF_FAT | GO:0005096:GTPase activator activity | 43 | 2.95E-04 |  |
| GO_BP_FAT | GO:0046578:regulation of Ras protein signal transduction | 40 | 1.13E-03 |  |
| GO_ MF_FAT | GO:0005089:Rho guanyl-nucleotide exchange factor activity | 20 | 1.15E-03 |  |
| GO_BP_FAT | GO:0035023:regulation of Rho protein signal transduction | 23 | 1.38E-03 |  |
| **Cluster 7** |  |  |  | **3.73** |
| GO_BP_FAT | GO:0001944:vasculature development | 57 | 3.51E-05 |  |
| GO_BP_FAT | GO:0001568:blood vessel development | 54 | 1.31E-04 |  |
| GO_BP_FAT | GO:0048514:blood vessel morphogenesis | 46 | 1.39E-04 |  |
| GO_BP_FAT | GO:0001525:angiogenesis | 31 | 1.91E-03 |  |
| **Cluster 8** |  |  |  | **3.72** |
| GO_BP_FAT | GO:0042330:taxis | 33 | 6.46E-06 |  |
| GO_BP_FAT | GO:0006935:chemotaxis | 33 | 6.46E-06 |  |
| GO_BP_FAT | GO:0007626:locomotory behavior | 49 | 1.63E-03 |  |
| GO_BP_FAT | GO:0007610:behavior | 69 | 1.90E-02 |  |
| **Cluster 9** |  |  |  | **3.68** |
| GO_BP_FAT | GO:0070663:regulation of leukocyte proliferation | 30 | 2.46E-07 |  |
| GO_BP_FAT | GO:0002694:regulation of leukocyte activation | 45 | 3.02E-07 |  |
| GO_BP_FAT | GO:0002684:positive regulation of immune system process | 55 | 3.25E-07 |  |
| GO_BP_FAT | GO:0050865:regulation of cell activation | 45 | 4.50E-07 |  |
| GO_BP_FAT | GO:0032944:regulation of mononuclear cell proliferation | 29 | 5.05E-07 |  |
| GO_BP_FAT | GO:0050670:regulation of lymphocyte proliferation | 29 | 5.05E-07 |  |
| GO_BP_FAT | GO:0050863:regulation of T cell activation | 34 | 1.76E-06 |  |
| GO_BP_FAT | GO:0042129:regulation of T cell proliferation | 23 | 2.48E-06 |  |
| GO_BP_FAT | GO:0051249:regulation of lymphocyte activation | 40 | 5.87E-06 |  |
| GO_BP_FAT | GO:0070665:positive regulation of leukocyte proliferation | 19 | 6.40E-05 |  |
| GO_BP_FAT | GO:0032946:positive regulation of mononuclear cell proliferation | 18 | 1.31E-04 |  |
| GO_BP_FAT | GO:0050671:positive regulation of lymphocyte proliferation | 18 | 1.31E-04 |  |
| GO_BP_FAT | GO:0002695:negative regulation of leukocyte activation | 18 | 2.92E-04 |  |
| GO_BP_FAT | GO:0050866:negative regulation of cell activation | 18 | 2.92E-04 |  |
| GO_BP_FAT | GO:0042102:positive regulation of T cell proliferation | 14 | 2.97E-04 |  |
| GO_BP_FAT | GO:0002683:negative regulation of immune system process | 22 | 5.96E-04 |  |
| GO_BP_FAT | GO:0051250:negative regulation of lymphocyte activation | 17 | 7.32E-04 |  |
| GO_BP_FAT | GO:0002696:positive regulation of leukocyte activation | 26 | 8.26E-04 |  |
| GO_BP_FAT | GO:0050867:positive regulation of cell activation | 26 | 1.13E-03 |  |
| GO_BP_FAT | GO:0050870:positive regulation of T cell activation | 19 | 1.68E-03 |  |
| GO_BP_FAT | GO:0050868:negative regulation of T cell activation | 14 | 2.12E-03 |  |
| GO_BP_FAT | GO:0050672:negative regulation of lymphocyte proliferation | 12 | 4.01E-03 |  |
| GO_BP_FAT | GO:0032945:negative regulation of mononuclear cell proliferation | 12 | 4.01E-03 |  |
| GO_BP_FAT | GO:0070664:negative regulation of leukocyte proliferation | 12 | 4.01E-03 |  |
| GO_BP_FAT | GO:0051251:positive regulation of lymphocyte activation | 23 | 4.54E-03 |  |
| GO_BP_FAT | GO:0030888:regulation of B cell proliferation | 11 | 6.25E-03 |  |
| GO_BP_FAT | GO:0042130:negative regulation of T cell proliferation | 10 | 7.62E-03 |  |
| GO_BP_FAT | GO:0050864:regulation of B cell activation | 14 | 2.37E-02 |  |
| GO_BP_FAT | GO:0030890:positive regulation of B cell proliferation | 7 | 5.94E-02 |  |
| GO_BP_FAT | GO:0050871:positive regulation of B cell activation | 9 | 1.04E-01 |  |
| GO_BP_FAT | GO:0030889:negative regulation of B cell proliferation | 4 | 1.34E-01 |  |
| **Cluster 10** |  |  |  | **3.30** |
| GO_ MF_FAT | GO:0005125:cytokine activity | 41 | 2.89E-04 |  |
| GO_ MF_FAT | GO:0008009:chemokine activity | 14 | 5.67E-04 |  |
| GO_ MF_FAT | GO:0042379:chemokine receptor binding | 14 | 7.52E-04 |  |
| **Cluster 11** |  |  |  | **3.26** |
| GO_BP_FAT | GO:0002684:positive regulation of immune system process | 55 | 3.25E-07 |  |
| GO_BP_FAT | GO:0048584:positive regulation of response to stimulus | 45 | 6.13E-05 |  |
| GO_BP_FAT | GO:0002764:immune response-regulating signal transduction | 18 | 2.25E-04 |  |
| GO_BP_FAT | GO:0050778:positive regulation of immune response | 34 | 2.96E-04 |  |
| GO_BP_FAT | GO:0002768:immune response-regulating cell surface receptor signaling pathway | 16 | 3.11E-04 |  |
| GO_BP_FAT | GO:0002253:activation of immune response | 24 | 5.56E-04 |  |
| GO_BP_FAT | GO:0002429:immune response-activating cell surface receptor signaling pathway | 14 | 1.66E-03 |  |
| GO_BP_FAT | GO:0002757:immune response-activating signal transduction | 15 | 2.71E-03 |  |
| GO_BP_FAT | GO:0050851:antigen receptor-mediated signaling pathway | 11 | 1.51E-02 |  |
| GO_BP_FAT | GO:0050852:T cell receptor signaling pathway | 6 | 1.54E-01 |  |
| **Cluster 12** |  |  |  | **3.12** |
| GO_ MF_FAT | GO:0032561:guanyl ribonucleotide binding | 69 | 6.74E-04 |  |
| GO_ MF_FAT | GO:0019001:guanyl nucleotide binding | 69 | 6.74E-04 |  |
| GO_ MF_FAT | GO:0005525:GTP binding | 67 | 9.33E-04 |  |
| **Cluster 13** |  |  |  | **2.91** |
| GO_ MF_FAT | GO:0043167:ion binding | 579 | 1.29E-05 |  |
| GO_ MF_FAT | GO:0046872:metal ion binding | 567 | 1.67E-05 |  |
| GO_ MF_FAT | GO:0043169:cation binding | 571 | 1.93E-05 |  |
| GO_ MF_FAT | GO:0046914:transition metal ion binding | 325 | 7.42E-01 |  |
| GO_ MF_FAT | GO:0008270:zinc ion binding | 254 | 8.82E-01 |  |
| **Cluster 14** |  |  |  | **2.72** |
| GO_ MF_FAT | GO:0046983:protein dimerization activity | 70 | 5.42E-05 |  |
| GO_ MF_FAT | GO:0042802:identical protein binding | 52 | 1.07E-02 |  |
| GO_ MF_FAT | GO:0042803:protein homodimerization activity | 37 | 1.22E-02 |  |
| **Cluster 15** |  |  |  | **2.70** |
| GO_ MF_FAT | GO:0019899:enzyme binding | 48 | 6.14E-04 |  |
| GO_ MF_FAT | GO:0019901:protein kinase binding | 19 | 2.95E-03 |  |
| GO_ MF_FAT | GO:0019900:kinase binding | 21 | 4.53E-03 |  |
| **Cluster 16** |  |  |  | **2.52** |
| GO_BP_FAT | GO:0006468:protein amino acid phosphorylation | 118 | 1.02E-04 |  |
| GO_ MF_FAT | GO:0004672:protein kinase activity | 106 | 1.38E-04 |  |
| GO_ MF_FAT | GO:0017076:purine nucleotide binding | 287 | 3.34E-04 |  |
| GO_BP_FAT | GO:0006796:phosphate metabolic process | 149 | 3.59E-04 |  |
| GO_BP_FAT | GO:0006793:phosphorus metabolic process | 149 | 3.59E-04 |  |
| GO_ MF_FAT | GO:0032555:purine ribonucleotide binding | 276 | 4.21E-04 |  |
| GO_ MF_FAT | GO:0032553:ribonucleotide binding | 276 | 4.21E-04 |  |
| GO_BP_FAT | GO:0016310:phosphorylation | 122 | 2.03E-03 |  |
| GO_ MF_FAT | GO:0030554:adenyl nucleotide binding | 223 | 2.14E-02 |  |
| GO_ MF_FAT | GO:0001883:purine nucleoside binding | 224 | 2.45E-02 |  |
| GO_ MF_FAT | GO:0032559:adenyl ribonucleotide binding | 212 | 2.52E-02 |  |
| GO_ MF_FAT | GO:0001882:nucleoside binding | 225 | 2.64E-02 |  |
| GO_ MF_FAT | GO:0000166:nucleotide binding | 307 | 3.41E-02 |  |
| GO_ MF_FAT | GO:0005524:ATP binding | 207 | 3.99E-02 |  |
| GO_ MF_FAT | GO:0004674:protein serine/threonine kinase activity | 62 | 1.62E-01 |  |
| **Cluster 17** |  |  |  | **2.48** |
| GO_BP_FAT | GO:0043068:positive regulation of programmed cell death | 56 | 6.90E-05 |  |
| GO_BP_FAT | GO:0010942:positive regulation of cell death | 56 | 8.65E-05 |  |
| GO_BP_FAT | GO:0043065:positive regulation of apoptosis | 54 | 2.02E-04 |  |
| GO_BP_FAT | GO:0042981:regulation of apoptosis | 101 | 4.67E-04 |  |
| GO_BP_FAT | GO:0043067:regulation of programmed cell death | 102 | 4.86E-04 |  |
| GO_BP_FAT | GO:0010941:regulation of cell death | 102 | 5.91E-04 |  |
| GO_BP_FAT | GO:0006917:induction of apoptosis | 34 | 1.02E-02 |  |
| GO_BP_FAT | GO:0012502:induction of programmed cell death | 34 | 1.02E-02 |  |
| GO_BP_FAT | GO:0043069:negative regulation of programmed cell death | 36 | 3.16E-01 |  |
| GO_BP_FAT | GO:0060548:negative regulation of cell death | 36 | 3.25E-01 |  |
| GO_BP_FAT | GO:0043066:negative regulation of apoptosis | 35 | 3.38E-01 |  |
| **Cluster 18** |  |  |  | **2.30** |
| GO_BP_FAT | GO:0009967:positive regulation of signal transduction | 45 | 7.74E-06 |  |
| GO_BP_FAT | GO:0010647:positive regulation of cell communication | 47 | 1.97E-05 |  |
| GO_BP_FAT | GO:0010740:positive regulation of protein kinase cascade | 21 | 9.27E-03 |  |
| GO_BP_FAT | GO:0043123:positive regulation of I-kappaB kinase/NF-kappaB cascade | 10 | 2.33E-02 |  |
| GO_BP_FAT | GO:0043122:regulation of I-kappaB kinase/NF-kappaB cascade | 11 | 3.10E-02 |  |
| GO_BP_FAT | GO:0010627:regulation of protein kinase cascade | 29 | 4.89E-02 |  |
| GO_BP_FAT | GO:0043410:positive regulation of MAPKKK cascade | 11 | 8.16E-02 |  |
| GO_BP_FAT | GO:0043408:regulation of MAPKKK cascade | 18 | 9.80E-02 |  |
| **Cluster 19** |  |  |  | **2.27** |
| GO_BP_FAT | GO:0001775:cell activation | 55 | 8.51E-05 |  |
| GO_BP_FAT | GO:0045321:leukocyte activation | 48 | 4.09E-04 |  |
| GO_BP_FAT | GO:0048534:hemopoietic or lymphoid organ development | 57 | 8.52E-04 |  |
| GO_BP_FAT | GO:0002520:immune system development | 59 | 9.80E-04 |  |
| GO_BP_FAT | GO:0046649:lymphocyte activation | 41 | 1.72E-03 |  |
| GO_BP_FAT | GO:0030097:hemopoiesis | 48 | 7.33E-03 |  |
| GO_BP_FAT | GO:0042110:T cell activation | 25 | 1.52E-02 |  |
| GO_BP_FAT | GO:0002521:leukocyte differentiation | 27 | 4.88E-02 |  |
| GO_BP_FAT | GO:0030098:lymphocyte differentiation | 22 | 6.74E-02 |  |
| GO_BP_FAT | GO:0030217:T cell differentiation | 16 | 6.81E-02 |  |
| GO_BP_FAT | GO:0042113:B cell activation | 16 | 8.19E-02 |  |
| **Cluster 20** |  |  |  | **2.25** |
| GO_ MF_FAT | GO:0015171:amino acid transmembrane transporter activity | 16 | 1.06E-03 |  |
| GO_ MF_FAT | GO:0015294:solute:cation symporter activity | 19 | 4.07E-03 |  |
| GO_ MF_FAT | GO:0015293:symporter activity | 29 | 4.45E-03 |  |
| GO_ MF_FAT | GO:0005275:amine transmembrane transporter activity | 17 | 4.83E-03 |  |
| GO_ MF_FAT | GO:0015370:solute:sodium symporter activity | 11 | 6.20E-02 |  |
| **Cluster 21** |  |  |  | **2.23** |
| GO_ MF_FAT | GO:0019842:vitamin binding | 32 | 9.35E-05 |  |
| GO_ MF_FAT | GO:0070279:vitamin B6 binding | 15 | 6.68E-03 |  |
| GO_ MF_FAT | GO:0030170:pyridoxal phosphate binding | 15 | 6.68E-03 |  |
| GO_ MF_FAT | GO:0048037:cofactor binding | 33 | 2.94E-01 |  |
| **Cluster 22** |  |  |  | **2.10** |
| GO_BP_FAT | GO:0055074:calcium ion homeostasis | 27 | 1.67E-04 |  |
| GO_BP_FAT | GO:0055065:metal ion homeostasis | 29 | 1.85E-04 |  |
| GO_BP_FAT | GO:0048878:chemical homeostasis | 72 | 3.91E-04 |  |
| GO_BP_FAT | GO:0006875:cellular metal ion homeostasis | 27 | 4.05E-04 |  |
| GO_BP_FAT | GO:0050801:ion homeostasis | 60 | 4.79E-04 |  |
| GO_BP_FAT | GO:0006874:cellular calcium ion homeostasis | 25 | 5.32E-04 |  |
| GO_BP_FAT | GO:0055080:cation homeostasis | 41 | 8.17E-04 |  |
| GO_BP_FAT | GO:0042592:homeostatic process | 102 | 2.06E-03 |  |
| GO_BP_FAT | GO:0006873:cellular ion homeostasis | 52 | 2.20E-03 |  |
| GO_BP_FAT | GO:0055082:cellular chemical homeostasis | 52 | 3.87E-03 |  |
| GO_BP_FAT | GO:0055066:di-, tri-valent inorganic cation homeostasis | 32 | 4.33E-03 |  |
| GO_BP_FAT | GO:0007204:elevation of cytosolic calcium ion concentration | 12 | 5.10E-03 |  |
| GO_BP_FAT | GO:0019725:cellular homeostasis | 63 | 5.31E-03 |  |
| GO_BP_FAT | GO:0051480:cytosolic calcium ion homeostasis | 12 | 7.97E-03 |  |
| GO_BP_FAT | GO:0030003:cellular cation homeostasis | 32 | 7.97E-03 |  |
| GO_BP_FAT | GO:0030005:cellular di-, tri-valent inorganic cation homeostasis | 29 | 8.11E-03 |  |
| GO_BP_FAT | GO:0048015:phosphoinositide-mediated signaling | 10 | 7.15E-02 |  |
| GO_BP_FAT | GO:0010518:positive regulation of phospholipase activity | 7 | 1.17E-01 |  |
| GO_BP_FAT | GO:0007202:activation of phospholipase C activity | 7 | 1.17E-01 |  |
| GO_BP_FAT | GO:0010863:positive regulation of phospholipase C activity | 7 | 1.17E-01 |  |
| GO_BP_FAT | GO:0060193:positive regulation of lipase activity | 7 | 1.35E-01 |  |
| GO_BP_FAT | GO:0010517:regulation of phospholipase activity | 7 | 1.53E-01 |  |
| GO_BP_FAT | GO:0007200:activation of phospholipase C activity by G-protein coupled receptor protein signaling pathway coupled to IP3 second messenger | 6 | 2.25E-01 |  |
| GO_BP_FAT | GO:0060191:regulation of lipase activity | 7 | 2.38E-01 |  |
| GO_BP_FAT | GO:0051345:positive regulation of hydrolase activity | 14 | 2.63E-01 |  |
| **Cluster 23** |  |  |  | **2.07** |
| GO_BP_FAT | GO:0002053:positive regulation of mesenchymal cell proliferation | 10 | 1.14E-03 |  |
| GO_BP_FAT | GO:0010464:regulation of mesenchymal cell proliferation | 10 | 1.66E-03 |  |
| GO_BP_FAT | GO:0050679:positive regulation of epithelial cell proliferation | 9 | 4.30E-02 |  |
| GO_BP_FAT | GO:0050678:regulation of epithelial cell proliferation | 14 | 6.38E-02 |  |
| **Cluster 24** |  |  |  | **2.07** |
| GO_BP_FAT | GO:0051270:regulation of cell motion | 25 | 5.55E-03 |  |
| GO_BP_FAT | GO:0030334:regulation of cell migration | 22 | 7.41E-03 |  |
| GO_BP_FAT | GO:0040012:regulation of locomotion | 24 | 1.53E-02 |  |
| **Cluster 25** |  |  |  | **2.05** |
| GO_BP_FAT | GO:0006633:fatty acid biosynthetic process | 23 | 5.78E-04 |  |
| GO_BP_FAT | GO:0033559:unsaturated fatty acid metabolic process | 12 | 5.10E-03 |  |
| GO_BP_FAT | GO:0016053:organic acid biosynthetic process | 30 | 8.93E-03 |  |
| GO_BP_FAT | GO:0046394:carboxylic acid biosynthetic process | 30 | 8.93E-03 |  |
| GO_BP_FAT | GO:0006631:fatty acid metabolic process | 36 | 1.49E-02 |  |
| GO_BP_FAT | GO:0008610:lipid biosynthetic process | 45 | 1.44E-01 |  |

^1^ Each row corresponds to a Functional Annotation Tool (FAT) GO category inside a cluster.

^2^ GO terms inside each cluster.

Table D. Enriched Gene Set Enrichment Analysis categories encompassing transcript isoforms under-expressed (FDR-adjusted P-value < 0.05 and > 10 transcript isoforms) and over- expressed (Nominal P-value < 0.01 and > 10 transcript isoforms) in microglia relative to macrophages in the wild type mice.

| Categories | NG^1^ | P-value^2^ | FDR ^3^ |
| --- | --- | --- | --- |
| **Under-expressed in microglia relative to macrophages** | | | |
| KEGGKEGG_Ribosome | 64 | 0.00E+00 | 0.00E+00 |
| Structural_Constituent_Of_Ribosome | 55 | 0.00E+00 | 0.00E+00 |
| KEGGKEGG_Hematopoietic_Cell_Lineage | 46 | 0.00E+00 | 0.00E+00 |
| Immune_Response | 123 | 0.00E+00 | 0.00E+00 |
| KEGGKEGG_Primary_Immunodeficiency | 19 | 0.00E+00 | 0.00E+00 |
| Immune_System_Process | 175 | 0.00E+00 | 3.27E-03 |
| Locomotory_Behavior | 47 | 0.00E+00 | 5.08E-03 |
| Chemokine_Receptor_Binding | 20 | 0.00E+00 | 5.28E-03 |
| Chemokine_Activity | 20 | 0.00E+00 | 7.10E-03 |
| Cytokine_Activity | 43 | 0.00E+00 | 7.61E-03 |
| Translation | 98 | 0.00E+00 | 7.40E-03 |
| KEGGKEGG_Leishmania_Infection | 31 | 0.00E+00 | 2.29E-02 |
| Behavior | 57 | 0.00E+00 | 2.31E-02 |
| Hydrolase_Activity_Acting_On_Carbon_Nitrogen_Not_Peptidebonds | 21 | 2.02E-03 | 2.21E-02 |
| G_Protein_Coupled_Receptor_Binding | 26 | 0.00E+00 | 2.30E-02 |
| KEGGKEGG_B_Cell_Receptor_Signaling_Pathway | 49 | 0.00E+00 | 2.56E-02 |
| Programmed_Cell_Death | 224 | 0.00E+00 | 3.04E-02 |
| Response_To_Chemical_Stimulus | 150 | 0.00E+00 | 2.91E-02 |
| Apoptosis_Go | 223 | 0.00E+00 | 2.76E-02 |
| KEGGKEGG_Aminoacyl_Trna_Biosynthesis | 20 | 0.00E+00 | 2.73E-02 |
| KEGGKEGG_Intestinal_Immune_Network_For_Iga_Production | 22 | 0.00E+00 | 3.07E-02 |
| Positive_Regulation_Of_Immune_Response | 15 | 3.87E-03 | 3.21E-02 |
| Regulation_Of_Apoptosis | 181 | 0.00E+00 | 3.42E-02 |
| KEGGKEGG_Cytokine_Cytokine_Receptor_Interaction | 131 | 0.00E+00 | 3.46E-02 |
| KEGG_Chemokine_Signaling_Pathway | 112 | 0.00E+00 | 3.80E-02 |
| Amine_Transmembrane_Transporter_Activity | 23 | 8.46E-03 | 3.79E-02 |
| Regulation_Of_Programmed_Cell_Death | 182 | 0.00E+00 | 3.92E-02 |
| RNA_Binding | 143 | 0.00E+00 | 4.12E-02 |
| KEGG_Spliceosome | 62 | 0.00E+00 | 4.23E-02 |
| **Over-expressed in microglia relative to macrophages** | | | |
| Enzyme_Linked_Receptor_Protein_Signaling_Pathway | 82 | 0.00E+00 | 4.06E-02 |
| Extracellular_Matrix_Structural_Constituent | 14 | 0.00E+00 | 9.83E-02 |
| Transforming_Growth_Factor_Beta_Receptor_Signaling_Pathway | 26 | 0.00E+00 | 1.27E-01 |
| G_Protein_Coupled_Receptor_Activity | 42 | 0.00E+00 | 1.38E-01 |
| Anatomical_Structure_Morphogenesis | 174 | 0.00E+00 | 2.00E-01 |
| KEGG_Tight_Junction | 64 | 0.00E+00 | 2.07E-01 |
| Neurite_Development | 28 | 1.92E-03 | 1.43E-01 |
| Transmembrane_Receptor_Protein_Serine_Threonine_Kinase_Signaling_Pathway | 32 | 2.02E-03 | 7.55E-02 |
| Pattern_Specification_Process | 13 | 2.09E-03 | 1.35E-01 |
| Transmembrane_Receptor_Protein_Tyrosine_Kinase_Signaling_Pathway | 47 | 2.11E-03 | 2.15E-01 |
| KEGG_Abc_Transporters | 21 | 4.18E-03 | 1.40E-01 |
| Transmembrane_Receptor_Protein_Kinase_Activity | 30 | 5.94E-03 | 1.76E-01 |
| Amino_Sugar_Metabolic_Process | 12 | 6.01E-03 | 1.25E-01 |
| Neuron_Development | 33 | 9.94E-03 | 2.31E-01 |

^1^ NG: number of genes in each enriched category.

^2^ P-value: Nominal P-value.

^3^ FDR: adjusted P-value.

Table E. Genes exhibiting an alternative splicing event between microglia and macrophages in wild type mice including at least two transcript isoforms and at least one over- or under-expressed (FDR-adjusted P-value < 0.05) transcript isoforms between cell types.

| Gene Symbol | Gene name | Differential expression | | |
| --- | --- | --- | --- | --- |
|  |  | Over^1^ | Under^2^ | Non^3^ |
| Nf1 | Neurofibromatosis 1 | 1 | 0 | 6 |
| Foxj1 | Forkhead box J1 | 0 | 2 | 2 |
| Zfp869 | Zinc finger protein 869 | 1 | 1 | 7 |
| Hspa12a | Heat shock protein 12A | 1 | 0 | 5 |
| Tmpo | Thymopoietin | 0 | 1 | 9 |
| Abr | Active BCR-related gene | 1 | 1 | 3 |
| Brd9 | Bromodomain containing 9 | 0 | 1 | 4 |
| Esco1 | Establishment of cohesion 1 homolog 1 | 1 | 0 | 4 |
| Hmg20b | High mobility group 20B | 0 | 1 | 4 |
| Tm7sf2 | Transmembrane 7 superfamily member 2 | 0 | 1 | 4 |
| Fxc1 | Translocase of inner mitochondrial membrane 10B | 0 | 1 | 6 |
| Ccdc28b | Coiled coil domain containing 28B | 2 | 0 | 2 |
| Ndst1 | N-deacetylase/N-sulfotransferase (heparan glucosaminyl) 1 | 1 | 1 | 2 |
| AK154275 | Predicted gene 4673 | 1 | 0 | 3 |
| Asap1 | ArfGAP with SH3 domain, ankyrin repeat and PH domain1 | 0 | 1 | 3 |
| Pcyt2 | Phosphate cytidylyltransferase 2, ethanolamine | 0 | 1 | 3 |
| Cul5 | Cullin 5 | 1 | 0 | 3 |
| Dlgap4 | Discs, large homolog-associated protein 4 | 1 | 0 | 3 |
| Fam175a | Family with sequence similarity 175, member A | 1 | 0 | 3 |
| Hus1 | Hus1 homolog (S. pombe) | 0 | 1 | 3 |
| Itga1 | Integrin alpha 1 | 0 | 1 | 3 |
| Mcph1 | Microcephaly, primary autosomal recessive 1 | 1 | 0 | 3 |
| Olfm1 | Olfactomedin 1 | 1 | 0 | 3 |
| Utp14b | UTP14, U3 small nucleolar ribonucleoprotein, homolog B | 1 | 0 | 3 |
| Rbm5 | RNA binding motif protein 5 | 1 | 1 | 4 |
| Rprd2 | Regulation of nuclear pre-mRNA domain containing 2 | 1 | 0 | 5 |
| Atp6v0a1 | ATPase, H+ transporting, lysosomal V0 subunit A1 | 0 | 1 | 5 |
| Sema4a | Sema domain, immunoglobulin domain (Ig), transmembrane domain (TM) and short cytoplasmic domain, (semaphorin) 4A | 1 | 0 | 5 |
| Zfp672 | Zinc finger protein 672 | 1 | 0 | 5 |
| Zfp26 | Zinc finger protein 26 | 0 | 1 | 7 |
| Mark3 | MAP/microtubule affinity-regulating kinase 3 | 1 | 1 | 1 |
| Nfrkb | Nuclear factor related to kappa B binding protein | 1 | 1 | 1 |
| Pcf11 | Cleavage and polyadenylation factor subunit homolog | 1 | 1 | 1 |
| 4932441J04Rik | RIKEN cDNA 4932441J04 gene | 1 | 0 | 2 |
| Gcap14 | Coiled-coil serine rich 2 | 1 | 0 | 2 |
| Lrrc29 | Leucine rich repeat containing 29 | 0 | 1 | 2 |
| Ubqln1 | Ubiquilin 1 | 1 | 0 | 2 |
| Zxdc | ZXD family zinc finger C | 1 | 0 | 2 |
| 2310015A10Rik | RIKEN cDNA 2310015A10 gene | 1 | 0 | 2 |
| 5730419I09Rik | C2 calcium-dependent domain containing 5 | 1 | 0 | 2 |
| Acbd6 | Acyl-Coenzyme A binding domain containing 6 | 1 | 0 | 2 |
| Acot7 | Acyl-CoA thioesterase 7 | 0 | 1 | 2 |
| Akap7 | A kinase (PRKA) anchor protein 7 | 0 | 1 | 2 |
| Cbx5 | Chromobox 5 | 1 | 0 | 2 |
| Cdk9 | Cyclin-dependent kinase 9 (CDC2-related kinase) | 0 | 1 | 2 |
| Chkb | Choline kinase beta | 1 | 0 | 2 |
| Col18a1 | Collagen, type XVIII, alpha 1 | 1 | 0 | 2 |
| Eno1 | Enolase 1, alpha non-neuron | 1 | 0 | 2 |
| Ggps1 | Geranylgeranyl diphosphate synthase 1 | 1 | 0 | 2 |
| Golim4 | Golgi integral membrane protein 4 | 0 | 1 | 2 |
| Gpatch2 | G patch domain containing 2 | 0 | 1 | 2 |
| Kdm3a | Lysine (K)-specific demethylase 3A | 1 | 0 | 2 |
| Mkl2 | MKL/myocardin-like 2 | 1 | 0 | 2 |
| Myadm | Myeloid-associated differentiation marker | 1 | 0 | 2 |
| Ppm1h | Protein phosphatase 1H (PP2C domain containing) | 1 | 0 | 2 |
| Ppp2r5e | Protein phosphatase 2, regulatory subunit B', epsilon | 1 | 0 | 2 |
| Ptp4a3 | Protein tyrosine phosphatase 4a3 | 0 | 1 | 2 |
| Purg | Purine-rich element binding protein G | 0 | 1 | 2 |
| Rnf126 | Ring finger protein 126 | 1 | 0 | 2 |
| Saal1 | Serum amyloid A-like 1 | 0 | 1 | 2 |
| Setd1a | SET domain containing 1A | 1 | 0 | 2 |
| Skiv2l | Superkiller viralicidic activity 2-like | 0 | 1 | 2 |
| Smchd1 | SMC hinge domain containing 1 | 1 | 0 | 2 |
| Snrk | SNF related kinase | 0 | 1 | 2 |
| Snrnp70 | Small nuclear ribonucleoprotein 70 (U1) | 0 | 1 | 2 |
| Ube2m | Ubiquitin-conjugating enzyme E2M | 1 | 0 | 2 |
| Usp45 | Ubiquitin specific petidase 45 | 1 | 0 | 2 |
| Hivep3 | Human immunodeficiency virus type I enhancer binding protein 3 | 2 | 0 | 3 |
| Hps1 | Hermansky-Pudlak syndrome 1 homolog | 0 | 1 | 4 |
| Tmem134 | Transmembrane protein 134 | 1 | 0 | 4 |
| Dtx3l | Deltex 3-like | 0 | 1 | 4 |
| Efna4 | Ephrin A4 | 0 | 1 | 4 |
| Far2 | Fatty acyl CoA reductase 2 | 1 | 0 | 4 |
| Gng3 | Guanine nucleotide binding protein (G protein), gamma 3 | 0 | 1 | 4 |
| Lrrc49 | Leucine rich repeat containing 49 | 0 | 1 | 4 |
| Wdr12 | WD repeat domain 12 | 1 | 0 | 4 |
| Wdr67 | TBC1 domain family, member 31 | 1 | 0 | 4 |
| Dnm1l | Dynamin 1-like | 1 | 0 | 6 |
| Arhgap17 | Rho GTPase activating protein 17 | 1 | 1 | 7 |
| AK197085 | cDNA sequence BC023829 | 1 | 0 | 1 |
| Carns1 | Carnosine synthase 1 | 1 | 0 | 1 |
| Cdc123 | Cell division cycle 123 | 0 | 1 | 1 |
| Dyx1c1 | Dyslexia susceptibility 1 candidate 1 homolog | 0 | 1 | 1 |
| Eno2 | Enolase 2, gamma neuronal | 1 | 0 | 1 |
| Grpel2 | GrpE-like 2, mitochondrial | 1 | 0 | 1 |
| Lrp12 | Low density lipoprotein-related protein 12 | 0 | 1 | 1 |
| Sat1 | Spermidine/spermine N1-acetyl transferase 1 | 0 | 1 | 1 |
| Stoml1 | Stomatin-like 1 | 0 | 1 | 1 |
| Zfp622 | Zinc finger protein 622 | 0 | 1 | 1 |
| 9930104L06Rik | RIKEN cDNA 9930104L06 gene | 0 | 1 | 1 |
| Afg3l1 | AFG3-like AAA ATPase 1 | 1 | 0 | 1 |
| Alg13 | Asparagine-linked glycosylation 13 | 1 | 0 | 1 |
| Arid1b | AT rich interactive domain 1B (SWI-like) | 1 | 0 | 1 |
| Btbd19 | BTB (POZ) domain containing 19 | 0 | 1 | 1 |
| Ccdc138 | Coiled-coil domain containing 138 | 0 | 1 | 1 |
| Ccdc21 | Centrosomal protein 85 | 1 | 0 | 1 |
| Cyp26b1 | Cytochrome P450, family 26, subfamily b, polypeptide 1 | 1 | 0 | 1 |
| Dcun1d5 | DCN1, defective in cullin neddylation 1, domain containing 5 | 1 | 0 | 1 |
| Dnajc25 | DnaJ (Hsp40) homolog, subfamily C, member 25 | 0 | 1 | 1 |
| Dym | Dymeclin | 1 | 0 | 1 |
| Higd1a | HIG1 domain family, member 1A | 0 | 1 | 1 |
| Hsf2 | Heat shock factor 2 | 0 | 1 | 1 |
| Ifnar2 | Interferon (alpha and beta) receptor 2 | 1 | 0 | 1 |
| Kdm5b | Lysine (K)-specific demethylase 5B | 1 | 0 | 1 |
| Khdrbs1 | KH domain containing, RNA binding, signal transduction associated 1 | 1 | 0 | 1 |
| Klrk1 | Killer cell lectin-like receptor subfamily K, member 1 | 1 | 0 | 1 |
| Mrps15 | Mitochondrial ribosomal protein S15 | 1 | 0 | 1 |
| Ofcc1 | Orofacial cleft 1 candidate 1 | 1 | 0 | 1 |
| Prkcz | Protein kinase C, zeta | 1 | 0 | 1 |
| Ptpn13 | Protein tyrosine phosphatase, non-receptor type 13 | 1 | 0 | 1 |
| Scaf11 | SR-related CTD-associated factor 11 | 1 | 0 | 1 |
| Spg7 | Spastic paraplegia 7 homolog | 1 | 0 | 1 |
| Tpsab1 | Tryptase alpha/beta 1 | 1 | 0 | 1 |
| U2af1 | U2 small nuclear ribonucleoprotein auxiliary factor (U2AF) 1 | 0 | 1 | 1 |
| Slc25a25 | Solute carrier family 25 (mitochondrial carrier, phosphate carrier), member 25 | 1 | 1 | 2 |
| Rnf216 | Ring finger protein 216 | 0 | 1 | 3 |
| Smarcd3 | SWI/SNF related, matrix associated, actin dependent regulator of chromatin, subfamily d, member 3 | 1 | 0 | 3 |
| Tmem179b | Transmembrane protein 179B | 1 | 0 | 3 |
| AK079527 | Predicted gene 5106 | 1 | 0 | 3 |
| AK081414 | - | 1 | 0 | 3 |
| Acbd5 | Acyl-Coenzyme A binding domain containing 5 | 1 | 0 | 3 |
| Azin1 | Antizyme inhibitor 1 | 1 | 0 | 3 |
| Ehbp1l1 | EH domain binding protein 1-like 1 | 0 | 1 | 3 |
| Kdm4b | Lysine (K)-specific demethylase 4B | 1 | 0 | 3 |
| Meaf6 | MYST/Esa1-associated factor 6 | 1 | 0 | 3 |
| Mxi1 | Max interacting protein 1 | 0 | 1 | 3 |
| Srrm1 | Serine/arginine repetitive matrix 1 | 0 | 1 | 3 |
| Tpm3 | Tropomyosin 3, gamma | 1 | 0 | 3 |
| Yipf6 | Yip1 domain family, member 6 | 0 | 1 | 3 |
| Zfp935 | Zinc finger protein 935 | 0 | 1 | 3 |
| Cep57 | Centrosomal protein 57 | 1 | 0 | 5 |
| Slc4a7 | Solute carrier family 4, sodium bicarbonate cotransporter, member 7 | 1 | 1 | 6 |
| Zmynd8 | Zinc finger, MYND-type containing 8 | 1 | 0 | 7 |
| Vps53 | Vacuolar protein sorting 53 | 0 | 1 | 11 |
| Ctps2 | Cytidine 5'-triphosphate synthase 2 | 1 | 1 | 1 |
| Trim11 | Tripartite motif-containing 11 | 1 | 1 | 1 |
| Zdhhc20 | Zinc finger, DHHC domain containing 20 | 1 | 1 | 1 |
| Kcnk13 | Potassium channel, subfamily K, member 13 | 1 | 0 | 2 |
| Tmem41b | Transmembrane protein 41B | 0 | 1 | 2 |
| Arhgap24 | Rho GTPase activating protein 24 | 1 | 0 | 2 |
| Asb7 | Ankyrin repeat and SOCS box-containing 7 | 0 | 1 | 2 |
| Btf3l4 | Basic transcription factor 3-like 4 | 0 | 1 | 2 |
| Cyp2r1 | Cytochrome P450, family 2, subfamily r, polypeptide 1 | 0 | 1 | 2 |
| Dcaf11 | DDB1 and CUL4 associated factor 11 | 1 | 0 | 2 |
| Diap3 | Diaphanous homolog 3 | 1 | 0 | 2 |
| Dusp14 | Dual specificity phosphatase 14 | 0 | 1 | 2 |
| Fau | Finkel-Biskis-Reilly murine sarcoma virus (FBR-MuSV) ubiquitously expressed (fox derived) | 1 | 0 | 2 |
| Mbd4 | Methyl-CpG binding domain protein 4 | 1 | 0 | 2 |
| Mettl16 | Methyltransferase like 16 | 1 | 0 | 2 |
| Nfx1 | Nuclear transcription factor, X-box binding 1 | 1 | 0 | 2 |
| Pisd | Phosphatidylserine decarboxylase | 1 | 0 | 2 |
| Prkcsh | Protein kinase C substrate 80K-H | 1 | 0 | 2 |
| Psd4 | Pleckstrin and Sec7 domain containing 4 | 1 | 0 | 2 |
| Tial1 | Tia1 cytotoxic granule-associated RNA binding protein-like 1 | 0 | 1 | 2 |
| Trio | Triple functional domain (PTPRF interacting) | 0 | 1 | 2 |
| Wbscr22 | Williams Beuren syndrome chromosome region 22 | 1 | 0 | 2 |
| Zfand6 | Zinc finger, AN1-type domain 6 | 1 | 0 | 2 |
| Hmox2 | Heme oxygenase (decycling) 2 | 1 | 1 | 3 |
| Tmem188 | CTD nuclear envelope phosphatase 1 regulatory subunit 1 | 1 | 0 | 4 |
| Tnrc6b | Trinucleotide repeat containing 6b | 1 | 0 | 4 |
| Ahcyl2 | S-adenosylhomocysteine hydrolase-like 2 | 1 | 0 | 4 |
| Brwd1 | Bromodomain and WD repeat domain containing 1 | 1 | 0 | 4 |
| Celf1 | CUGBP, Elav-like family member 1 | 1 | 0 | 4 |
| Cep250 | Centrosomal protein 250 | 1 | 0 | 4 |
| Rnasel | Ribonuclease L (2', 5'-oligoisoadenylate synthetase-dependent) | 1 | 0 | 4 |
| Tmem161a | Transmembrane protein 161A | 0 | 1 | 4 |
| AK043531 | H2A histone family, member Y | 2 | 2 | 3 |
| Wdr33 | WD repeat domain 33 | 1 | 0 | 6 |
| Clk1 | CDC-like kinase 1 | 1 | 1 | 2 |
| 4933421E11Rik | Ligand dependent nuclear receptor interacting factor 1 | 1 | 0 | 3 |
| Hnrnpd | Heterogeneous nuclear ribonucleoprotein D | 1 | 0 | 3 |
| Men1 | Multiple endocrine neoplasia 1 | 1 | 0 | 3 |
| Nemf | Nuclear export mediator factor | 1 | 0 | 3 |
| Sec31a | Sec31 homolog A | 1 | 0 | 3 |
| Tbp | TATA binding protein | 1 | 0 | 3 |
| Urgcp | Upregulator of cell proliferation | 0 | 1 | 3 |
| Gas2l1 | Growth arrest-specific 2 like 1 | 1 | 1 | 4 |
| Rad52 | RAD52 homolog | 2 | 0 | 4 |
| Zfp207 | Zinc finger protein 207 | 0 | 1 | 5 |
| Hnrnpa2b1 | Heterogeneous nuclear ribonucleoprotein A2/B1 | 1 | 0 | 5 |
| Tex264 | Testis expressed gene 264 | 0 | 1 | 5 |
| Acin1 | Apoptotic chromatin condensation inducer 1 | 1 | 0 | 9 |
| Ganab | Alpha glucosidase 2 alpha neutral subunit | 1 | 0 | 4 |
| Phf11 | PHD finger protein 11 | 2 | 1 | 1 |
| Mdm4 | Transformed mouse 3T3 cell double minute 4 | 0 | 1 | 6 |
| Srsf7 | Serine/arginine-rich splicing factor 7 | 0 | 1 | 6 |
| Fubp1 | Far upstream element (FUSE) binding protein 1 | 1 | 0 | 8 |
| Fam132b | Family with sequence similarity 132, member B | 1 | 1 | 4 |
| Elf2 | E74-like factor 2 | 1 | 1 | 5 |
| 4732471D19Rik | SUMO-interacting motifs containing 1 | 0 | 1 | 6 |
| Cox16 | Cytochrome c oxidase assembly protein 16 | 1 | 0 | 8 |
| Akr1c18 | Aldo-keto reductase family 1, member C18 | 1 | 0 | 1 |
| Nvl | Nuclear VCP-like | 1 | 0 | 1 |
| Ttc19 | Tetratricopeptide repeat domain 19 | 1 | 0 | 10 |
| AK038933 | Predicted gene 16070 | 0 | 1 | 1 |
| Cys1 | Cystin 1 | 0 | 1 | 1 |
| Gjb6 | Gap junction protein, beta 6 | 0 | 1 | 1 |
| Hhatl | Hedgehog acyltransferase-like | 0 | 1 | 1 |
| Slc47a1 | Solute carrier family 47, member 1 | 0 | 1 | 1 |
| Smn1 | Survival motor neuron 1 | 0 | 1 | 1 |
| Snap91 | Synaptosomal-associated protein 91 | 0 | 1 | 1 |
| Zic4 | Zinc finger protein of the cerebellum 4 | 0 | 1 | 1 |
| Crlf3 | Cytokine receptor-like factor 3 | 0 | 1 | 2 |
| Myl6 | Myosin, light polypeptide 6, alkali, smooth muscle and non-muscle | 0 | 1 | 2 |
| Arid4a | AT rich interactive domain 4A (RBP1-like) | 0 | 1 | 2 |
| Ly6h | Lymphocyte antigen 6 complex, locus H | 0 | 1 | 2 |
| Nme5 | NME/NM23 family member 5 | 0 | 1 | 2 |
| Gnb1l | Guanine nucleotide binding protein (G protein), beta polypeptide 1-like | 0 | 1 | 3 |
| Mybph | Myosin binding protein H | 0 | 1 | 3 |
| Sox17 | SRY (sex determining region Y)-box 17 | 0 | 1 | 4 |

1Over: transcript isoforms over-expressed in macrophage

2Under: transcript isoforms under-expressed in macrophage

3Non: not differentially expressed transcript isoforms (FDR-adjusted P-value < 0.05)

Table F. Enriched (DAVID Enrichment score ES > 2) functional clusters of categories encompassing transcript isoforms expressed solely in macrophages or in microglia in wild type mice.

| Cell type and Category^1^ | Terms^2^ | Gene Count | P-value | ES |
| --- | --- | --- | --- | --- |
| **Macrophages** | | | | |
| Cluster 1 |  |  |  | 2.39 |
| GO_ MF_FAT | GO:0004252:serine-type endopeptidase activity | 4 | 5.67E-04 |  |
| GO_ MF_FAT | GO:0008236:serine-type peptidase activity | 4 | 7.92E-04 |  |
| GO_ MF_FAT | GO:0017171:serine hydrolase activity | 4 | 8.03E-04 |  |
| GO_ MF_FAT | GO:0004175:endopeptidase activity | 4 | 5.61E-03 |  |
| GO_ MF_FAT | GO:0070011:peptidase activity, acting on L-amino acid peptides | 4 | 1.50E-02 |  |
| GO_ MF_FAT | GO:0008233:peptidase activity | 4 | 1.69E-02 |  |
| GO_BP_FAT | GO:0006508:proteolysis | 4 | 3.52E-02 |  |
| **Microglia** | | | | |
| Cluster 1 |  |  |  | 4.18 |
| GO_ MF_FAT | GO:0022803:passive transmembrane transporter activity | 24 | 1.91E-09 |  |
| GO_ MF_FAT | GO:0015267:channel activity | 24 | 1.91E-09 |  |
| GO_ MF_FAT | GO:0022838:substrate specific channel activity | 23 | 7.76E-09 |  |
| GO_ MF_FAT | GO:0005216:ion channel activity | 22 | 2.29E-08 |  |
| GO_BP_FAT | GO:0006811:ion transport | 29 | 4.24E-07 |  |
| GO_ MF_FAT | GO:0022836:gated channel activity | 17 | 2.42E-06 |  |
| GO_ MF_FAT | GO:0005261:cation channel activity | 15 | 1.16E-05 |  |
| GO_ MF_FAT | GO:0022832:voltage-gated channel activity | 12 | 4.77E-05 |  |
| GO_ MF_FAT | GO:0005244:voltage-gated ion channel activity | 12 | 4.77E-05 |  |
| GO_ MF_FAT | GO:0046873:metal ion transmembrane transporter activity | 15 | 6.55E-05 |  |
| GO_ MF_FAT | GO:0022843:voltage-gated cation channel activity | 9 | 4.66E-04 |  |
| GO_BP_FAT | GO:0006812:cation transport | 17 | 1.92E-03 |  |
| GO_BP_FAT | GO:0030001:metal ion transport | 15 | 3.07E-03 |  |
| GO_BP_FAT | GO:0055085:transmembrane transport | 15 | 4.38E-03 |  |
| GO_ MF_FAT | GO:0005267:potassium channel activity | 7 | 1.00E-02 |  |
| GO_ MF_FAT | GO:0005249:voltage-gated potassium channel activity | 6 | 1.32E-02 |  |
| GO_BP_FAT | GO:0006813:potassium ion transport | 7 | 2.28E-02 |  |
| GO_BP_FAT | GO:0015672:monovalent inorganic cation transport | 10 | 2.37E-02 |  |
| GO_ MF_FAT | GO:0031420:alkali metal ion binding | 8 | 2.73E-02 |  |
| GO_ MF_FAT | GO:0030955:potassium ion binding | 5 | 8.64E-02 |  |
| Cluster 2 |  |  |  | 3.05 |
| GO_BP_FAT | GO:0048878:chemical homeostasis | 16 | 1.51E-04 |  |
| GO_BP_FAT | GO:0042391:regulation of membrane potential | 9 | 2.28E-04 |  |
| GO_BP_FAT | GO:0055082:cellular chemical homeostasis | 13 | 3.23E-04 |  |
| GO_BP_FAT | GO:0006873:cellular ion homeostasis | 12 | 9.43E-04 |  |
| GO_BP_FAT | GO:0050801:ion homeostasis | 12 | 2.36E-03 |  |
| GO_BP_FAT | GO:0019725:cellular homeostasis | 13 | 2.71E-03 |  |
| GO_BP_FAT | GO:0042592:homeostatic process | 17 | 6.49E-03 |  |
| Cluster 3 |  |  |  | 2.54 |
| GO_BP_FAT | GO:0007267:cell-cell signaling | 17 | 2.38E-06 |  |
| GO_BP_FAT | GO:0019226:transmission of nerve impulse | 14 | 1.39E-05 |  |
| GO_BP_FAT | GO:0007268:synaptic transmission | 10 | 7.70E-04 |  |
| GO_BP_FAT | GO:0046903:secretion | 11 | 9.38E-04 |  |
| GO_BP_FAT | GO:0003001:generation of a signal involved in cell-cell signaling | 7 | 1.02E-03 |  |
| GO_BP_FAT | GO:0046879:hormone secretion | 5 | 2.32E-03 |  |
| GO_BP_FAT | GO:0009914:hormone transport | 5 | 2.53E-03 |  |
| GO_BP_FAT | GO:0032940:secretion by cell | 9 | 4.09E-03 |  |
| GO_BP_FAT | GO:0010817:regulation of hormone levels | 7 | 8.31E-03 |  |
| GO_BP_FAT | GO:0030072:peptide hormone secretion | 3 | 7.86E-02 |  |
| GO_BP_FAT | GO:0002790:peptide secretion | 3 | 8.68E-02 |  |
| GO_BP_FAT | GO:0015833:peptide transport | 3 | 1.35E-01 |  |
| GO_BP_FAT | GO:0006887:exocytosis | 4 | 1.91E-01 |  |

^1^ Each row corresponds to a Functional Annotation Tool (FAT) GO category inside a cluster.

^2^ GO terms inside each cluster.

Table G. Differentially expressed genes (FDR-adjusted P-value < 0.05) between wild type (WT) and IDO1-knockout (KO) mice within cell type, including log_2_(fold ratio).

| Gene Symbol | Gene name | Log_2_(WT/ IDO1-KO) |
| --- | --- | --- |
| **Microglia** | | |
| Bcas1 | breast carcinoma amplified sequence 1 | 2.31951 |
| Kif5a | kinesin family member 5A | 2.25243 |
| Mbp | myelin basic protein | 2.10829 |
| Mobp | myelin-associated oligodendrocytic basic protein | 1.8695 |
| Ifnb1 | interferon beta 1, fibroblast | 1.63948 |
| Mid1 | midline 1 | 1.61391 |
| Klk6 | kallikrein related-peptidase 6 | 1.45666 |
| Plekhb1 | pleckstrin homology domain containing, family B (evectins) member 1 | 1.34113 |
| Prr18 | proline rich 18 | 1.22566 |
| Unc13c | unc-13 homolog C | 1.18053 |
| Kif5c | kinesin family member 5C | 1.17307 |
| Gjb1 | gap junction protein, beta 1 | 1.1064 |
| S100b | S100 protein, beta polypeptide, neural | 1.09079 |
| Hapln2 | hyaluronan and proteoglycan link protein 2 | 1.05284 |
| Olig1 | oligodendrocyte transcription factor 1 | 1.04173 |
| Stmn4 | stathmin-like 4 | 1.03925 |
| Tmem151a | transmembrane protein 151A | 1.03658 |
| Mog | myelin oligodendrocyte glycoprotein | 1.03153 |
| Opalin | oligodendrocytic myelin paranodal and inner loop protein | 1.02265 |
| Tmem125 | transmembrane protein 125 | 1.01846 |
| Tubb4a | tubulin, beta 4A class IVA | 0.972721 |
| Olig2 | oligodendrocyte transcription factor 2 | 0.959413 |
| Cldn11 | claudin 11 | 0.954688 |
| Map6d1 | MAP6 domain containing 1 | 0.943816 |
| Plp1 | proteolipid protein (myelin) 1 | 0.943353 |
| Spock1 | sparc/osteonectin, cwcv and kazal-like domains proteoglycan 1 | 0.940586 |
| Nkain1 | Na+/K+ transporting ATPase interacting 1 | 0.933082 |
| Rell1 | RELT-like 1 | 0.933034 |
| Tmem88b | transmembrane protein 88B | 0.929952 |
| Gpr37 | G protein-coupled receptor 37 | 0.925465 |
| Sox10 | SRY (sex determining region Y)-box 10 | 0.89782 |
| Lgi3 | leucine-rich repeat LGI family, member 3 | 0.894414 |
| Jph4 | junctophilin 4 | 0.885661 |
| Fa2h | fatty acid 2-hydroxylase | 0.879607 |
| Tubb3 | tubulin, beta 3 class III | 0.872596 |
| Il33 | interleukin 33 | 0.871807 |
| Kcna6 | potassium voltage-gated channel, shaker-related, subfamily, member 6 | 0.869428 |
| Cntn2 | contactin 2 | 0.848411 |
| Mapt | microtubule-associated protein tau | 0.839774 |
| Lrrn1 | leucine rich repeat protein 1, neuronal | 0.838271 |
| Efnb3 | ephrin B3 | 0.834591 |
| Ermn | ermin, ERM-like protein | 0.814256 |
| Dusp26 | dual specificity phosphatase 26 (putative) | 0.811775 |
| Gjc3 | gap junction protein, gamma 3 | 0.800036 |
| Pllp | plasma membrane proteolipid | 0.798515 |
| Aspa | aspartoacylase | 0.785977 |
| Kcna1 | potassium voltage-gated channel, shaker-related subfamily, member 1 | 0.777877 |
| Hcn2 | hyperpolarization-activated, cyclic nucleotide-gated K+ 2 | 0.772187 |
| Cmtm6 | CKLF-like MARVEL transmembrane domain containing 6 | 0.771987 |
| Sez6l2 | seizure related 6 homolog like 2 | 0.766402 |
| Adamts4 | a disintegrin-like and metallopeptidase (reprolysin type) with thrombospondin type 1 motif, 4 | 0.760244 |
| Fez1 | fasciculation and elongation protein zeta 1 (zygin I) | 0.757503 |
| Tmem229a | transmembrane protein 229A | 0.749488 |
| Efhd1 | EF hand domain containing 1 | 0.742199 |
| Srcin1 | SRC kinase signaling inhibitor 1 | 0.740682 |
| Hba-a2 | hemoglobin alpha, adult chain 2 | 0.730706 |
| Sept4 | septin 4 | 0.727578 |
| Slc15a2 | solute carrier family 15 (H+/peptide transporter), member 2 | 0.712033 |
| Slain1 | SLAIN motif family, member 1 | 0.694269 |
| Kndc1 | kinase non-catalytic C-lobe domain (KIND) containing 1 | 0.689484 |
| Ptprd | protein tyrosine phosphatase, receptor type, D | 0.673478 |
| Beta-s, Hbb-bs | hemoglobin, beta adult s chain | 0.668602 |
| 1810011O10Rik | RIKEN cDNA 1810011O10 gene | 0.657675 |
| Hbb-b1 | hemoglobin, beta adult major chain | 0.618422 |
| Ppp1r16b | protein phosphatase 1, regulatory (inhibitor) subunit 16B | 0.593925 |
| Mal, Mpv17 | myelin and lymphocyte protein, T cell differentiation protein | 0.528305 |
| Aplp1 | amyloid beta (A4) precursor-like protein 1 | 0.477909 |
| Mag | myelin-associated glycoprotein | 0.451227 |
| AK140265 |  | 0.424154 |
| Ccr1 | chemokine (C-C motif) receptor 1 | -0.503946 |
| Oasl2 | 2'-5' oligoadenylate synthetase-like 2 | -0.507449 |
| Emilin2 | elastin microfibril interfacer 2 | -0.510399 |
| Hp | haptoglobin | -0.55794 |
| S100a6 | S100 calcium binding protein A6 (calcyclin) | -0.641146 |
| Cxcr2 | chemokine (C-X-C motif) receptor 2 | -0.644654 |
| Glipr2 | GLI pathogenesis-related 2 | -0.655396 |
| Ms4a4c | membrane-spanning 4-domains, subfamily A, member 4C | -0.661402 |
| Ifitm6 | interferon induced transmembrane protein 6 | -0.674877 |
| Fpr2, Fpr-rs2 | formyl peptide receptor 2 | -0.773126 |
| Ly6c2 | lymphocyte antigen 6 complex, locus C2 | -0.781014 |
| 9930111J21Rik2 | RIKEN cDNA 9930111J21 gene 2 | -0.83463 |
| Slfn1 | schlafen 1 | -0.883383 |
| Zbp1 | Z-DNA binding protein 1 | -0.898679 |
| Irg1, Cad | immunoresponsive gene 1 | -0.94192 |
| Gbp2 | guanylate binding protein 2 | -0.976531 |
| Dynlt1a | dynein light chain Tctex-type 1A | -1.09003 |
| Oas3 | 2'-5' oligoadenylate synthetase 3 | -1.14147 |
| Myoc | myocilin | -1.19444 |
| Upp1 | uridine phosphorylase 1 | -1.4118 |
| Asprv1 | aspartic peptidase, retroviral-like 1 | -1.42676 |
| Cfb | complement factor B | -1.44362 |
| Lrg1 | leucine-rich alpha-2-glycoprotein 1 | -1.44589 |
| Ly6i | lymphocyte antigen 6 complex, locus I | -1.49055 |
| F10 | coagulation factor X | -1.54624 |
| Plac8 | placenta-specific 8 | -1.58291 |
| Ifitm1 | interferon induced transmembrane protein 1 | -1.61129 |
| Cga | glycoprotein hormones, alpha subunit | -1.71623 |
| Pomc | pro-opiomelanocortin-alpha | -1.88438 |
| Steap4 | STEAP family member 4 | -2.28338 |
| Sele | selectin, endothelial cell | -2.52165 |
| Saa3 | serum amyloid A 3 | -3.31996 |
| Darc | atypical chemokine receptor 1 (Duffy blood group) | -4.21202 |
| Slc15a2 | solute carrier family 15 (H+/peptide transporter), member 2 | 1.02185 |
| AF032968 |  | 0.910461 |
| Rpl39 | ribosomal protein L39 | 0.683357 |
| Apoc1 | apolipoprotein C-I | 0.499185 |
| Diras2 | DIRAS family, GTP-binding RAS-like 2 | 0.430628 |
| Rell1 | RELT-like 1 | 0.394837 |
| AI607873 | expressed sequence AI607873 | -0.408308 |
| 3110043O21Rik | RIKEN cDNA 3110043O21 gene | -0.424056 |
| Denr | density-regulated protein | -0.425078 |
| Cdc42ep3 | CDC42 effector protein (Rho GTPase binding) 3 | -0.427249 |
| Dab2 | disabled 2, mitogen-responsive phosphoprotein | -0.428076 |
| Lrrc25 | leucine rich repeat containing 25 | -0.432013 |
| Rnf149 | ring finger protein 149 | -0.440681 |
| Serpinb1a | serine (or cysteine) peptidase inhibitor, clade B, member 1a | -0.440809 |
| Ms4a6d | membrane-spanning 4-domains, subfamily A, member 6D | -0.447812 |
| Mcpt4 | mast cell protease 4 | -0.453525 |
| Fabp4 | fatty acid binding protein 4, adipocyte | -0.457382 |
| Mtmr10 | myotubularin related protein 10 | -0.458651 |
| Rabgef1 | RAB guanine nucleotide exchange factor (GEF) 1 | -0.468681 |
| Sgms2 | sphingomyelin synthase 2 | -0.471271 |
| Fam198b | family with sequence similarity 198, member B | -0.481518 |
| Fabp7 | fatty acid binding protein 7, brain | -0.484375 |
| Nr4a3 | nuclear receptor subfamily 4, group A, member 3 | -0.486816 |
| Spred2 | sprouty-related, EVH1 domain containing 2 | -0.491944 |
| Il2rb | interleukin 2 receptor, beta chain | -0.494466 |
| Hk1 | hexokinase 1 | -0.496041 |
| Map3k6 | mitogen-activated protein kinase kinase kinase 6 | -0.505601 |
| Raph1 | Ras association (RalGDS/AF-6) and pleckstrin homology domains 1 | -0.50607 |
| Car13 | carbonic anhydrase 13 | -0.508375 |
| Tmtc2 | transmembrane and tetratricopeptide repeat containing 2 | -0.516123 |
| Irs2 | insulin receptor substrate 2 | -0.519234 |
| Ell2 | elongation factor RNA polymerase II 2 | -0.520129 |
| Mdm2 | transformed mouse 3T3 cell double minute 2 | -0.52368 |
| Chsy3 | chondroitin sulfate synthase 3 | -0.529572 |
| Peli1 | pellino 1 | -0.530077 |
| Col18a1 | collagen, type XVIII, alpha 1 | -0.532345 |
| Gja1 | gap junction protein, alpha 1 | -0.551029 |
| Itk | IL2 inducible T cell kinase | -0.552151 |
| Cdk1 | cyclin-dependent kinase 1 | -0.569789 |
| Ppfibp1 | PTPRF interacting protein, binding protein 1 (liprin beta 1) | -0.570935 |
| Il6st | interleukin 6 signal transducer (oncostatin M receptor) | -0.574248 |
| Maff | v-maf musculoaponeurotic fibrosarcoma oncogene family, protein F (avian) | -0.574938 |
| Pdgfa | platelet derived growth factor, alpha | -0.579277 |
| Adarb1 | adenosine deaminase, RNA-specific, B1 | -0.579901 |
| Batf | basic leucine zipper transcription factor, ATF-like | -0.582655 |
| Cd209a | CD209a antigen | -0.583251 |
| Tmem2 | transmembrane protein 2 | -0.592894 |
| Cish | cytokine inducible SH2-containing protein | -0.597133 |
| Hilpda | hypoxia inducible lipid droplet associated | -0.60093 |
| Vcam1 | vascular cell adhesion molecule 1 | -0.601169 |
| Pls3 | plastin 3 (T-isoform) | -0.613584 |
| Gfpt2 | glutamine fructose-6-phosphate transaminase 2 | -0.617881 |
| Dpysl3 | dihydropyrimidinase-like 3 | -0.623074 |
| Slc39a14 | solute carrier family 39 (zinc transporter), member 14 | -0.623419 |
| P2ry6 | pyrimidinergic receptor P2Y, G-protein coupled, 6 | -0.627337 |
| Ube2c | ubiquitin-conjugating enzyme E2C | -0.627589 |
| Hk2 | hexokinase 2 | -0.628917 |
| Niacr1 | hydroxycarboxylic acid receptor 2 | -0.649111 |
| Arg1 | arginase, liver | -0.653281 |
| Bach1 | BTB and CNC homology 1 | -0.657532 |
| Creb3l1 | cAMP responsive element binding protein 3-like 1 | -0.659339 |
| Muc1 | mucin 1, transmembrane | -0.735784 |
| Cd274 | CD274 antigen | -0.736441 |
| Procr | protein C receptor, endothelial | -0.741626 |
| Cd109 | CD109 antigen | -0.741908 |
| Ptges | prostaglandin E synthase | -0.761752 |
| Ckap2l | cytoskeleton associated protein 2-like | -0.78086 |
| Cmpk2 | cytidine monophosphate (UMP-CMP) kinase 2, mitochondrial | -0.800451 |
| Tnip3 | TNFAIP3 interacting protein 3 | -0.801418 |
| Slc7a2 | solute carrier family 7 (cationic amino acid transporter, y+ system), member 2 | -0.814024 |
| Ifit2 | interferon-induced protein with tetratricopeptide repeats 2 | -0.836908 |
| Serpine1 | serine (or cysteine) peptidase inhibitor, clade E, member 1 | -0.84992 |
| Rhou | ras homolog gene family, member U | -0.855325 |
| Gdf15 | growth differentiation factor 15 | -0.870927 |
| Arc | activity regulated cytoskeletal-associated protein | -0.892317 |
| 9330154J02Rik | RIKEN cDNA 9330154J02 | -0.907147 |
| Plat | plasminogen activator, tissue | -0.907668 |
| Kif1a | kinesin family member 1A | -0.918973 |
| P2ry2 | purinergic receptor P2Y, G-protein coupled 2 | -0.921321 |
| Sema3c | sema domain, immunoglobulin domain (Ig), short basic domain, secreted, (semaphorin) 3C | -0.935194 |
| Tnf | tumor necrosis factor | -0.950365 |
| Armcx6 | armadillo repeat containing, X-linked 6 | -0.95732 |
| Pdpn | podoplanin | -0.967963 |
| Ifi205 | interferon activated gene 205 | -0.970983 |
| AK033051 |  | -0.980102 |
| Phlda1 | pleckstrin homology-like domain, family A, member 1 | -0.985195 |
| Tnfaip6 | tumor necrosis factor alpha induced protein 6 | -0.998676 |
| Ppap2b | phosphatidic acid phosphatase type 2B | -1.01547 |
| Ereg | epiregulin | -1.02293 |
| Ccl2 | chemokine (C-C motif) ligand 2 | -1.03105 |
| Cxcl1 | chemokine (C-X-C motif) ligand 1 | -1.0372 |
| Dynlt1a | dynein light chain Tctex-type 1A | -1.03961 |
| Smim3 | small integral membrane protein 3 | -1.04504 |
| 4931440P22Rik | RIKEN cDNA 4931440P22 gene | -1.05963 |
| Ch25h | cholesterol 25-hydroxylase | -1.11404 |
| Edn1 | endothelin 1 | -1.11512 |
| Prok2 | prokineticin 2 | -1.18488 |
| Gm11110 | predicted gene 11110 | -1.18812 |
| Rnd1 | Rho family GTPase 1 | -1.20347 |
| Tnfsf14 | tumor necrosis factor (ligand) superfamily, member 14 | -1.21585 |
| Ptgs2 | prostaglandin-endoperoxide synthase 2 | -1.23965 |
| F3 | coagulation factor III | -1.25211 |
| Mx2 | MX dynamin-like GTPase 2 | -1.26361 |
| Slamf1 | signaling lymphocytic activation molecule family member 1 | -1.3301 |
| S100a9 | S100 calcium binding protein A9 (calgranulin B) | -1.34386 |
| Steap4 | STEAP family member 4 | -1.34444 |
| AK009121 |  | -1.38488 |
| Gm14023 | predicted gene 14023 | -1.40604 |
| Ccl3 | chemokine (C-C motif) ligand 3 | -1.42794 |
| Ccl5 | chemokine (C-C motif) ligand 5 | -1.45147 |
| Il13 | interleukin 13 | -1.46847 |
| Gzmb | granzyme B | -1.4749 |
| Hba-a2 | hemoglobin alpha, adult chain 2 | -1.48106 |
| Areg | amphiregulin | -1.487 |
| Ccrn4l | CCR4 carbon catabolite repression 4-like (S. cerevisiae) | -1.48888 |
| Il10 | interleukin 10 | -1.51638 |
| Hbegf | heparin-binding EGF-like growth factor | -1.52455 |
| Lif | leukemia inhibitory factor | -1.5645 |
| Art3 | ADP-ribosyltransferase 3 | -1.60061 |
| Beta-s, Hbb-bs | hemoglobin, beta adult s chain | -1.61749 |
| Lipg | lipase, endothelial | -1.64734 |
| Ifih1 | interferon induced with helicase C domain 1 | -1.66035 |
| Rsad2 | radical S-adenosyl methionine domain containing 2 | -1.74366 |
| Ccl7 | chemokine (C-C motif) ligand 7 | -1.75504 |
| Tulp4 | tubby like protein 4 | -1.76346 |
| Isg15 | ISG15 ubiquitin-like modifier | -1.92341 |
| Ptgs2os | prostaglandin-endoperoxide synthase 2, opposite strand | -1.97375 |
| Gm14446 | predicted gene 14446 | -2.05192 |
| Ccl4 | chemokine (C-C motif) ligand 4 | -2.13478 |
| Il19 | interleukin 19 | -2.35677 |
| Csf2 | colony stimulating factor 2 (granulocyte-macrophage) | -2.46113 |
| Ifnb1 | interferon beta 1, fibroblast | -2.64361 |
| Plet1 | placenta expressed transcript 1 | -2.64581 |
| Oasl1 | 2'-5' oligoadenylate synthetase-like 1 | -2.74147 |
| Sele | selectin, endothelial cell | -3.03502 |
| Csf3 | colony stimulating factor 3 (granulocyte) | -3.53356 |

Fig A. Distribution of expression levels expressed in fragments per kilobase of transcript per million mapped fragments (FPKMs) for 28,403 annotated genes with at least 5 reads per transcript in IDO1-knockout microglia (IDO1_KO_Brain), wild type microglia (Wild_Brain), IDO1-knockout macrophages (IDO1_KO_Peritoneo), and wild type macrophages (Wild_Peritoneo).
